# A Compositional Look at the Human Gastrointestinal Microbiome and Immune Activation Parameters in HIV Infected Subjects

**DOI:** 10.1371/journal.ppat.1003829

**Published:** 2014-02-20

**Authors:** Ece A. Mutlu, Ali Keshavarzian, John Losurdo, Garth Swanson, Basile Siewe, Christopher Forsyth, Audrey French, Patricia DeMarais, Yan Sun, Lars Koenig, Stephen Cox, Phillip Engen, Prachi Chakradeo, Rawan Abbasi, Annika Gorenz, Charles Burns, Alan Landay

**Affiliations:** 1 Division of Gastroenterology, Hepatology and Nutrition, Department of Medicine, Rush University Medical Center, Chicago, Illinois, United States of America; 2 Department of Pharmacology, The Graduate College, Rush University, Chicago, Illinois, United States of America; 3 Department of Physiology and Molecular Biophysics, Rush University Medical Center, Chicago, Illinois, United States of America; 4 Division of Pharmacology, Utrecht Institute for Pharmaceutical Sciences, Faculty of Science, Utrecht University, Utrecht, The Netherlands; 5 Department of Immunology/Microbiology, Rush University Medical Center, Chicago, Illinois, United States of America; 6 Department of Biochemistry, The Graduate College, Rush University, Chicago, Illinois, United States of America; 7 Ruth M. Rothstein CORE Center/Department of Medicine, Rush University Medical Center, Chicago, Illinois, United States of America; 8 Research and Testing Laboratory, LLC., Lubbock, Texas, United States of America; 9 Rush Medical College, Armour Academic Center, Chicago, Illinois, United States of America; Stanford University, United States of America

## Abstract

HIV progression is characterized by immune activation and microbial translocation. One factor that may be contributing to HIV progression could be a dysbiotic microbiome. We therefore hypothesized that the GI mucosal microbiome is altered in HIV patients and this alteration correlates with immune activation in HIV. 121 specimens were collected from 21 HIV positive and 22 control human subjects during colonoscopy. The composition of the lower gastrointestinal tract mucosal and luminal bacterial microbiome was characterized using 16S rDNA pyrosequencing and was correlated to clinical parameters as well as immune activation and circulating bacterial products in HIV patients on ART. The composition of the HIV microbiome was significantly different than that of controls; it was less diverse in the right colon and terminal ileum, and was characterized by loss of bacterial taxa that are typically considered commensals. In HIV samples, there was a gain of some pathogenic bacterial taxa. This is the first report characterizing the terminal ileal and colonic mucosal microbiome in HIV patients with next generation sequencing. Limitations include use of HIV-infected subjects on HAART therapy.

## Author Summary

Human immunodeficiency virus (HIV) infection related illness progresses despite the control of the virus itself by medications that stop the replication of the virus. This happens because the immune system gets activated. While the causes for such activation of the immune system are not exactly known, immune activation in HIV infection may be occurring as a result of bacteria or their products in the digestive tract. This study looks at the types of bacteria that reside in the lower intestinal tract in patients infected with human immunodeficiency virus, using state of the art sequencing technology, that can simultaneously look at thousands of bacteria. We have found that the bacteria at the end of the small bowel (an area also called the terminal ileum), at the right and left sides of the large intestine and in the stool is different in patients infected with the human immunodeficiency virus. HIV patients harbor more bacteria that have been linked to other human diseases and have been previously described as harmful. This finding is new and could open up a new frontier of study that could now pave the way to gain a deeper understanding of how the HIV causes illness.

## Introduction

Human immunodeficiency virus (HIV) infection is a chronic illness characterized by progressive CD4+ T cell loss. With the advent of highly active anti-retroviral therapy (HAART), HIV infection is controlled, resulting in reduced death from opportunistic infections. However, despite successful viral suppression, many HIV patients have persistent inflammation/immune activation resulting in the development of non-HIV comorbidities including cardiovascular disease, osteoporosis, neurocognitive decline, cancer, as well as increased mortality [1], [2].

The mechanism of persistent inflammation and immune activation in HIV patients whose viral loads are successfully suppressed by HAART is not fully understood. However, several studies suggest that immune activation could be a consequence of gut-triggered systemic inflammation and microbial translocation [3]: This is not surprising because one of the earliest and principal sites of inflammation/immune activation and resultant CD4+ T cell infection by HIV is the gastrointestinal (GI) tract [4]. In fact, the GI tract houses the largest mucosal immune system in the body, and is a major interface between immune cells and the environment, with its substantial surface area. It also contains a significant amount of microbial mass, collectively referred to as the gastrointestinal microbiome, the majority of which is bacterial and has an estimated metabolic activity comparable to a human liver.

Little is known about the human GI tract microbiome in disease states including HIV, despite the fact that current evidence points toward a potential central role for the GI tract microbiome in HIV progression. Studies in HIV-infected humans show that bacterial products are increased in the circulation of patients with HIV [5], and this can occur even at early stages of HIV infection, before peripheral CD4+ T cell depletion reaches levels that can lead to clinically apparent disease and opportunistic infections [6]. Furthermore, polymicrobial bacterial DNA is detected in the circulation even in patients with suppressed viral loads [7]. The source of this increase is thought to be gastrointestinal tract permeability, which in turn is influenced by the composition and the function of the gastrointestinal microbiome, and the microbial- immune cell interactions across the gastrointestinal epithelium.

Direct evidence of alterations in the composition of the gastrointestinal tract microbiome has also been found for some bacterial taxa: Current data indicate increases in commensals that can be pathogenic such as Pseudomonas in the feces of subjects with HIV infection; and decreases in mostly beneficial commensals such as Lactobacilli and Bifidobacteria [6].Furthermore, prebiotics have been tested in HIV in an effort to alter the microbiome to have a more favorable composition, resulting in reduced CD4+ T cell activation and improvement in NK cell activity [8].

On the contrary to the human observations, studies on animal models have not supported the presence of an alteration in the bacterial microbiome [3], [9]:In a simian model of HIV with colitis, the composition of the GI tract microbiome was altered with increases in *Campylobacter* due to the colitis resulting from the presence of SIV, however, no specific differences were found attributable to SIV infection itself [10]. Similarly, in a metagenomic study of the fecal virome in SIV-infected macaques, no major differences were noted when the bacterial sequences were examined [9]. Possible reasons for this discrepancy in findings in the SIV model and human HIV could be related to the SIV model itself, or to the study of fecal samples to date. It is theoretically plausible that mucosal samples could provide higher relevance to immune activation given the close proximity of the microbiota to the GI tract epithelium and immune cells at the mucosal surface. On the other hand, human studies in HIV inherently carry major limitations by nature of the subjects themselves being on antiretroviral therapy or having additional lifestyle factors that cannot be tightly controlled for. Nevertheless, HIV-infected human studies are expected to provide significant information as to the mechanisms at play in the clinical setting, and are needed to confirm or refute the findings observed in SIV-infected macaques.

To date, a detailed and comprehensive look at the GI tract microbiome, especially the ileal and colonic mucosal microbiome in relation to the fecal microbiome has not been reported in HIV -infected humans Major advances in next generation sequencing technologies now allow for a comprehensive and rapid evaluation of the GI tract microbiome in humans at a fraction of the effort and cost, compared to the weeks and months required for such a sequencing effort less than a decade ago. The advent of these technologies has brought about rapid characterization of the bacterial microbiome in the GI tract by sequencing of bacterial genes, such as the 16SrDNA that are used in the taxonomical classification of bacteria. Therefore, these technologies represent an opportunity to characterize the HIV associated GI tract microbiome.

We aimed to study the composition of the gastrointestinal tract mucosal and luminal bacterial microbiome using 16S rDNA sequencing and to correlate the microbiome to clinical parameters as well as immune activation and circulating bacterial products in HIV-infected patients. We hypothesized that the GI mucosal microbiome is altered in HIV-infected patients and such an alteration correlates with immune activation.

## Results

### Description of the data

We obtained samples from the terminal ileum (TI), the right colon (RC), the left colon (LC) during colonoscopy, as well as fecal samples from the colonic lumen (F). The sample sites are shown in Table 1. There were no statistically significant differences in terms of age, gender, and race among the HIV and control subjects (Table S1). The mean CD4 count was 425+/−259 among the HIV subjects (Table S2). The viral load was <75 for 17 out of 21 patients; the remaining four subjects had a mean viral load of 1571+/−2059 cps/ml (Table S2). In 18 out of 21 HIV-infected subjects, colonoscopy was performed for colon cancer screening; and the remaining three subjects had constipation (n = 1), minor rectal bleeding (n = 2), and intermittent loose stools (n = 1). 13/21 HIV subjects were MSM; four had confirmed heterosexual acquisition of HIV; and in 4 subjects, the mode of HIV acquisition was not known.

**Table 1:** Sample sites and numbers.

Sample Site	HIV (n = 56)	Controls (n = 65)
Terminal ileum (TI)	8	9
Right colon (RC)	16	17
Left colon (LC)	13	18
Fecal (F)	19	21

We obtained 1,079,589 raw sequences, and 322,061,108 raw bases with an average of 8849 sequences/sample at an average length of 298 bps/sequence in two separate runs on a 454 instrument, as described in the methods. After quality–filtering (also as described in the methods), 455,452 total sequences and an average of 3733 sequences per sample were available, that were denoised, >250 bp long, de-multiplexed, reverse-primer-truncated and chimera-filtered for the rest of the analysis. The sequences were rarified to the minimum number of high quality sequences in all samples and normalized by total count for the alpha and beta diversity analyses conducted below.

### HIV infection is associated with a decrease in microbial diversity in the terminal ileum and the colon

**Figure 1:**
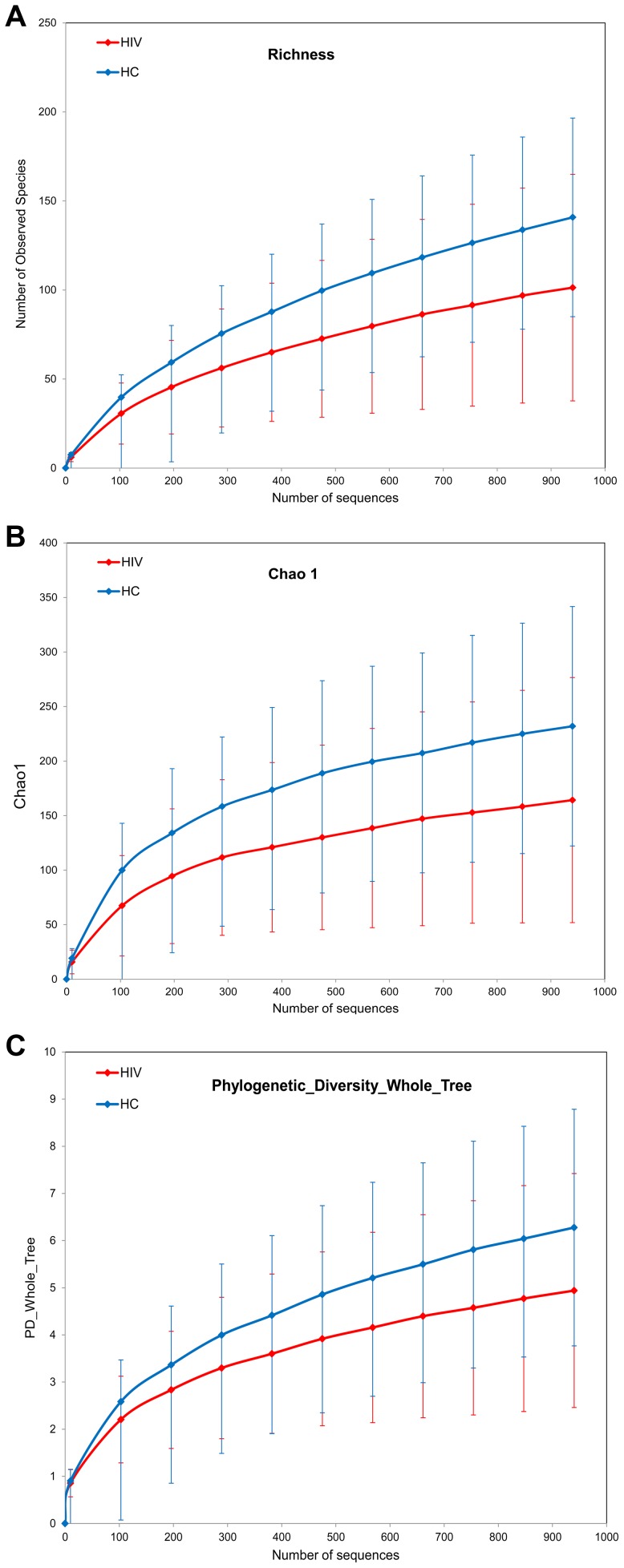
Diversity indices in HIV samples versus control samples. HIV samples have decreased diversity compared to controls. HIV samples are in red; Control samples are in blue. Panels : (a) OTU richness, (b) Chao1 index, (c) Phylogenetic Diversity (PD) Whole Tree metric.

**Figure 2:**
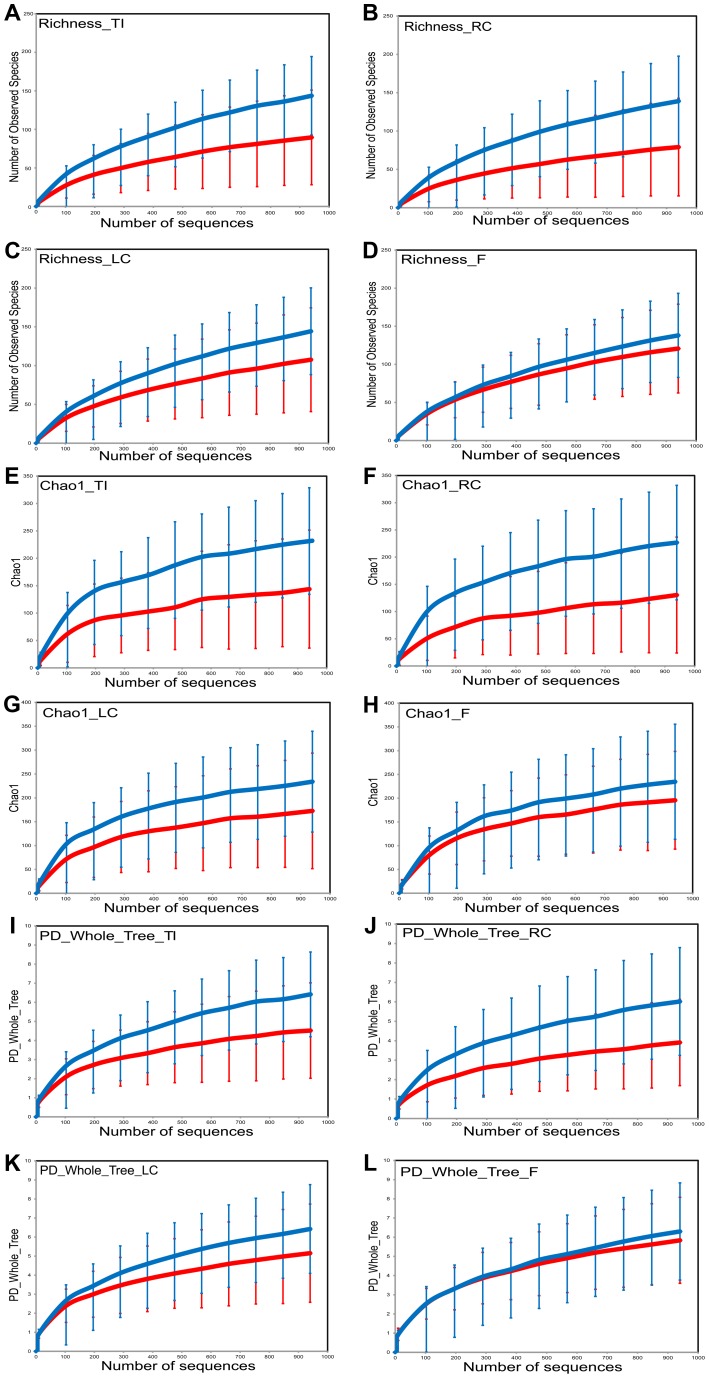
Sample diversity assessed by diversity indices in HIV cases versus controls by sample site. HIV cases have decreased diversity compared to controls. HIV samples are in red; Control samples are in blue. Diversity indices shown are OTU richness (panels (a–d)), Chao1 index (panels (e–h)), and Phylogenetic Diversity (PD) Whole Tree metric (panels (i–l)). Samples from ileum are shown in panels (a),(e),(i); samples from right colon are shown in panels (b),(f),(j) ; samples from left colon are shown in panels (c),(g),(k); and fecal samples are shown in panels (d),(h),(l).

Sample richness was assessed by operational taxonomic unit (OTU) counts in each individual sample: The number of observed OTUs in the HIV samples was less than that of the control samples (Figure 1a). When looked at by sample type, all sample types had less OTUs in the HIV group compared to the healthy controls. The differences between the HIV and controls groups were especially heightened for samples from the ileum and the right colon which showed numerically higher reductions in sample richness (Figure 2a–d).

Within sample bacterial diversity was also assessed by both the chao1 and the Phylogenetic Diversity (PD) Whole Tree metric. Both metrics demonstrated reduced overall diversity among the samples from HIV-infected subjects compared to controls (Figures 1b and 1c). The differences were also seen for each individual sample type, however samples from the mucosal surface of HIV-infected subjects were more divergent from those of controls, compared to the differences noted between fecal samples from the HIV and the control groups (Figures 2e–h, and 2i–l). Collectively, this data points towards a less diverse bacterial population in HIV throughout the TI and the colon as well as the luminal compartment, although the differences in diversity were most noticeable on the mucosal surfaces, and especially the right colon.

### The GI tract bacterial microbiome composition is significantly altered in HIV-infected subjects

**Figure 3:**
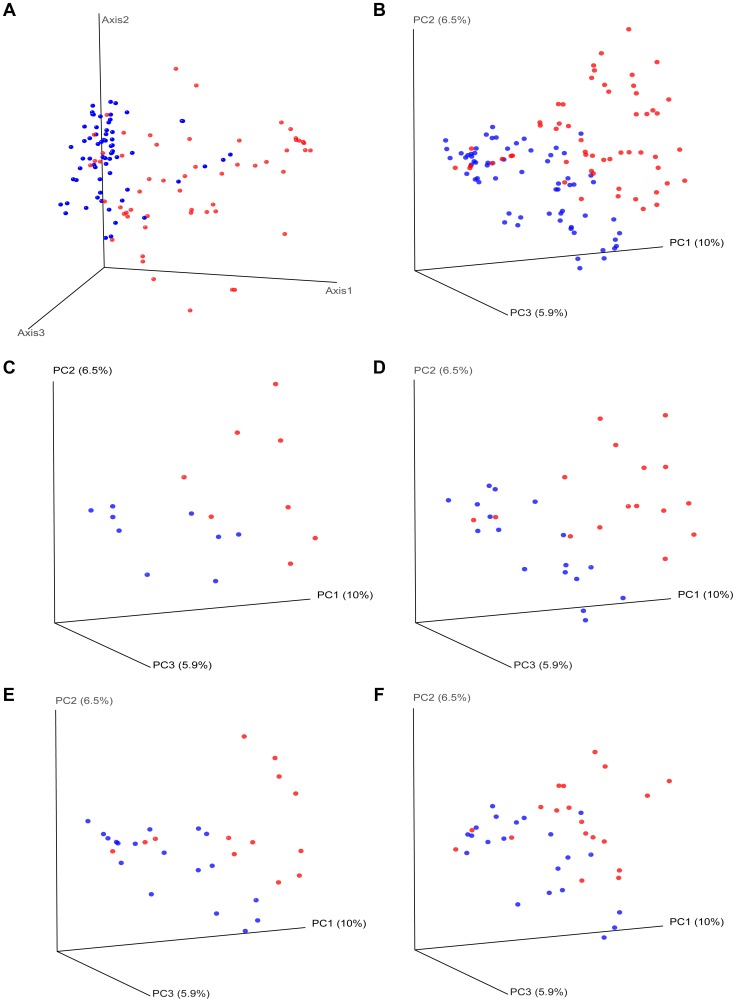
Beta diversity measures in HIV versus controls. HIV samples appear separated from control samples in beta diversity analyses. HIV samples are in red; Control samples are in blue. Panel (a) shows nonmetric multidimensional scaling (NMDS) of all the samples using the Bray-Curtis similarity at the OTU level. Panel (b) shows principal coordinates analysis of all of the samples using the Unifrac metric at the OTU level. Panels (c–f) show principal coordinates analysis using the Unifrac metric, by sample site. Panels: (c) For samples from ileum; (d) For samples from right colon; (e) For samples from left colon; (f) For fecal samples.

**Figure 4:**
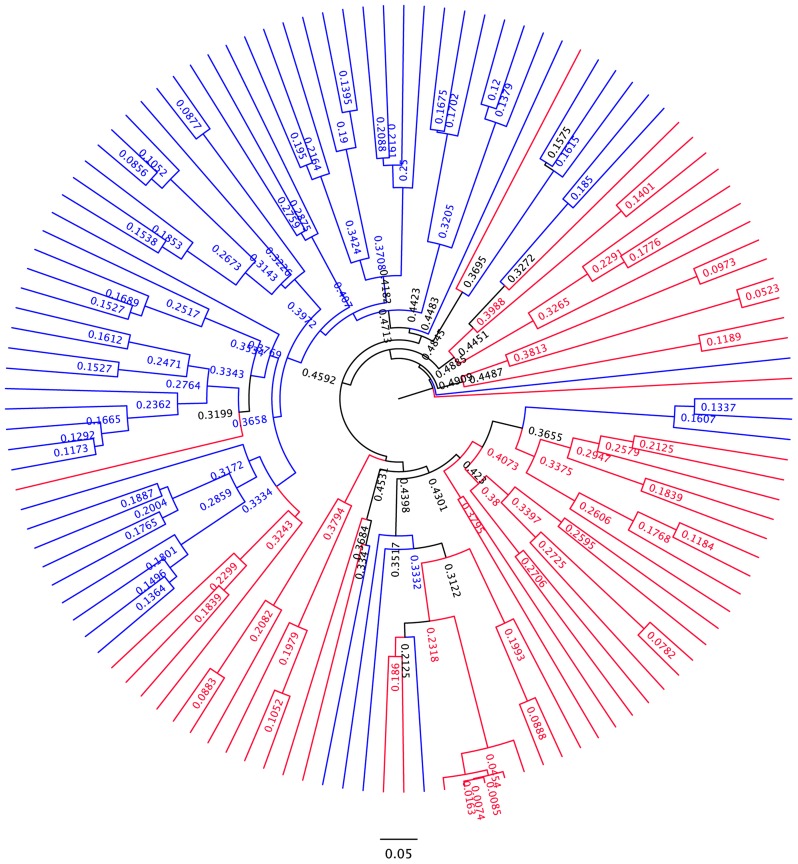
UPGMA dendogram based on Bray-Curtis similarity. Branches related to HIV samples are colored in red. Branches related to control samples are colored in blue. There is significant clustering within the dendogram.

In order to examine the global differences in bacterial composition between the HIV-infected subjects and the controls, we calculated distances between each sample using the Bray-Curtis similarity and unweighted Unifrac. We chose these two different measures for the following reason: The Bray-Curtis similarity is based on shared OTU counts between samples [11], whereas the Unifrac involves placing samples on a phylogenetic tree and calculating and summing branch lengths unique to a given sample on the tree [12]. Consequently, Bray-Curtis similarity gives equal importance to differences in every taxa, whereas in Unifrac, phylogenetically related taxa cause less divergent Unifrac values compared to distant or unrelated taxa which cause larger differences.

a **Differences in overall community composition in HIV-infected subjects vs. controls.** To uncover differences in bacterial community composition between the samples, we performed nonmetric multidimensional scaling (NMDS) of all the samples using the count-based Bray-Curtis similarity at the OTU level. NMDS is a powerful ordination method that can uncover nonlinear relationships between samples and is widely used in ecology. As shown in the results in Figure 3a, there was differential grouping of the control samples to the left of the graph, meanwhile samples from HIV-infected subjects were more dispersed than the control samples and were to the right. Hierarchical clustering of the samples with the unweighted pair group method with arithmetic mean (UPGMA) using the Bray-Curtis similarity additionally confirmed that samples from HIV –infected subjects clustered differently than those from controls (Figure 4). In order to test whether the visual differences seen with NMDS ordination and the clustering observed by UPGMA are significant, we compared global bacterial composition in the HIV and control groups at the genus level using perMANOVA with the Bray-Curtis similarity in a one-fixed-factor and one-level nested mixed random effects model (i.e. replicates within sample sites within disease presence (i.e. HIV vs. Controls)). We noted that the visual differences between HIV and controls were statistically significant (Table 2).

**Table 2:** PerMANOVA summary results.

Site	Model	Mean F*	P-value	Proportion of subsamplings rejecting the null hypothesis
**All**	Mixed Nested	10.491	0.0003	100%
**Ileum**	One-way	4.007	0.0010	100%
**Right Colon**	One-way	6.319	0.0002	100%
**Left Colon**	One-way	4.086	0.0004	100%
**Fecal**	One-way	3.418	0.0018	100%

*Mean F reflects the mean result of 1000 observed F values after 1000 subsamplings, each with 4999 randomizations.

To uncover relationships based on presence or absence of bacterial groups as well as their phylogenetic relatedness, we performed a principal coordinates analysis of the samples using the phylogenetic-tree-based Unifrac metric. As shown in Figure 3b, there was a different and tighter grouping of the control samples to the left of the graph, and HIV samples were again more dispersed and to the right. The overall Unifrac test was also statistically significant indicating clustering within the phylogenetic tree (p = 0.01).

b **Differences in overall community composition in HIV-infected subjects vs. controls by site of sampling.** We then compared samples from HIV-infected subjects and controls at each site (TI, RC, LC, F) using Unifrac based PCO ordination: Separation of samples was seen for each sample type, however the differential dispersion of samples was graphically more apparent for the TI and RC samples, with a few cases overlapping in the LC and F samples (Figure 3c–f). In order to assure that the findings we have observed are not an artifact of the sequence rarification and to get an estimate of the robustness of the results to our sequencing effort, we also performed jackknifed estimates of the Unifrac PCO analysis and the results obtained did not alter the conclusions (Figure S1, S2, S3, S4, S5).

We then also statistically compared global bacterial composition in the HIV and control groups at each site at the genus level using perMANOVA models. In all sites, differences between HIV and controls were statistically significant (Table 2).

c **Comparison of bacterial OTUs observed in HIV-infected subjects and controls with network analysis.** In order to evaluate bacterial OTUs associated with HIV and controls, we constructed a bipartite network of the bacterial OTUs and the samples, in which OTU-nodes are connected via edges to sample nodes in which their sequences are found. We have chosen the network analysis approach because it easily allows for visualization of thousands of OTUs in limited number of samples and can reveal significant associations. In the network, edge weights are determined by the number of sequences in an OTU; and clustering of the OTUs and samples are determined by a stochastic spring embedded algorithm as implemented in Cytoscape. The sample connections within the network are analyzed statistically with a G test of independence: Sample nodes from HIV-infected subjects and controls are tested to see whether they are more connected to a particular group than expected by chance. The network diagram is shown in Figure S6: In the center of the graph, sample nodes which are colored by HIV (in red) and controls (in blue) reveal significant clustering of the samples to either the right or the left of the graph, respectively for these groups. Some OTU nodes (shown in white) are only connected to HIV samples (in pink lines) while an equally numerous OTU nodes are only connected to the control samples (in lavender lines). OTU nodes that are connected to single samples lie in the periphery of the network, while those OTU nodes that are connected to multiple samples lie in the center of the network. The network statistics are shown in Table S3, and suggest an overall network with little interconnectedness overall, largely owing to the fact that the OTU nodes are only connected through the sample nodes. However, network statistics also indicate when the network is divided into two subnetworks (i.e. HIV network and control network), there are differences: The HIV node subnetwork has less average neighbors, more heterogeneity and less density compared to both the overall and the control node subnetwork. The HIV nodes are also farther apart from each other than the control nodes. These findings are compatible with the heterogeneity and disarray of the microbiome in HIV.

### HIV-infection is associated with increases in potentially pathogenic taxa in the GI tract microbiome

**Figure 5:**
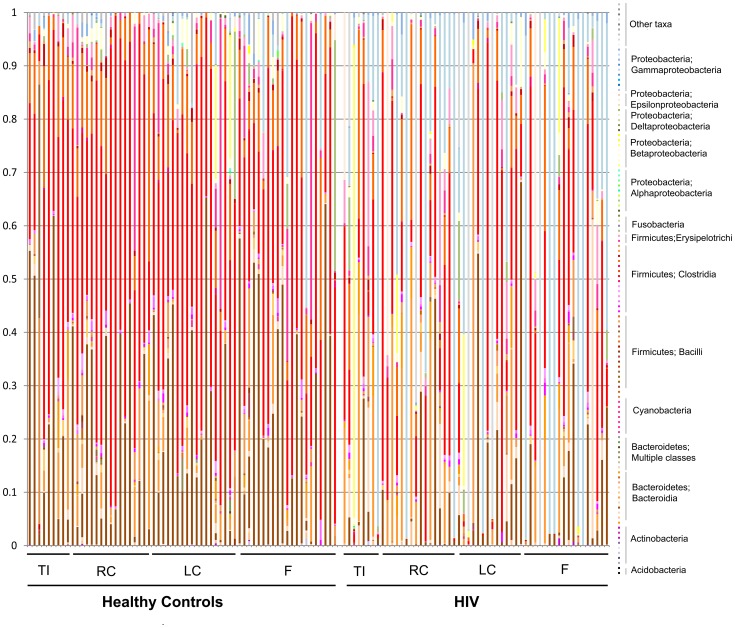
Stacked histogram of bacterial composition in all samples by disease and site, to the family level of taxonomic resolution. Each column represents bacterial composition in one single sample (n = 121 for all samples; n = 65 for controls; n = 56 for HIV). Y- axis denotes abundance of bacterial taxa as a percentage within the sample, with each column totaling 100%. X-axis shows samples from healthy subjects to the left of the graph, and samples from HIV subjects to the right of the graph. TI = samples from terminal ileum; RC = samples from right colon; LC = samples from left colon; F = fecal samples. While all identified families are shown on the graph, sample coloring is grouped by phylogeny. Among the colors used, Bacteroidales are shown in brown tones, Bacilli are shown in orange tones, Clostridia are shown in red tones, Alphaproteobacteria are shown in green tones, Betaproteobacteria are shown in yellow tones, Gammaproteobacteria are shown in blue tones. Major differences between control and HIV samples are visually apparent based on difference in coloring of the samples.

The mean and standard deviations of different bacterial taxa at each taxonomic level in the HIV and control samples are given in Tables S4, S5, S6, S7. Stacked histogram for all samples at a phylogenetic resolution at the family level is given in Figure 5. The histogram shows clear visual differences between the abundance of bacterial taxa in then control samples to the right (that have a lot of brown coloring) and HIV samples to the left (that have an increased abundance of Gamma Proteobacteria shown as blue coloring)

**Figure 6:**
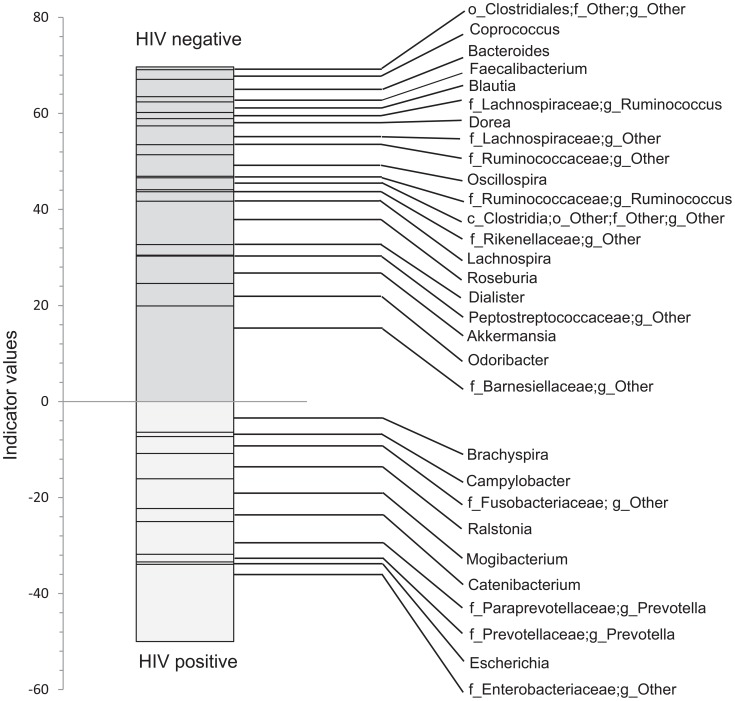
Indicator values for bacterial taxa, which are indicative of control or HIV samples. Indicator species analysis was performed after rarification and log transformation. Analysis was blocked by site. Genera and those unclassified bacterial members of families that not able to be classified down to a particular genus, that also have indicator values >15 and p<0.05 are shown.

**Figure 7:**
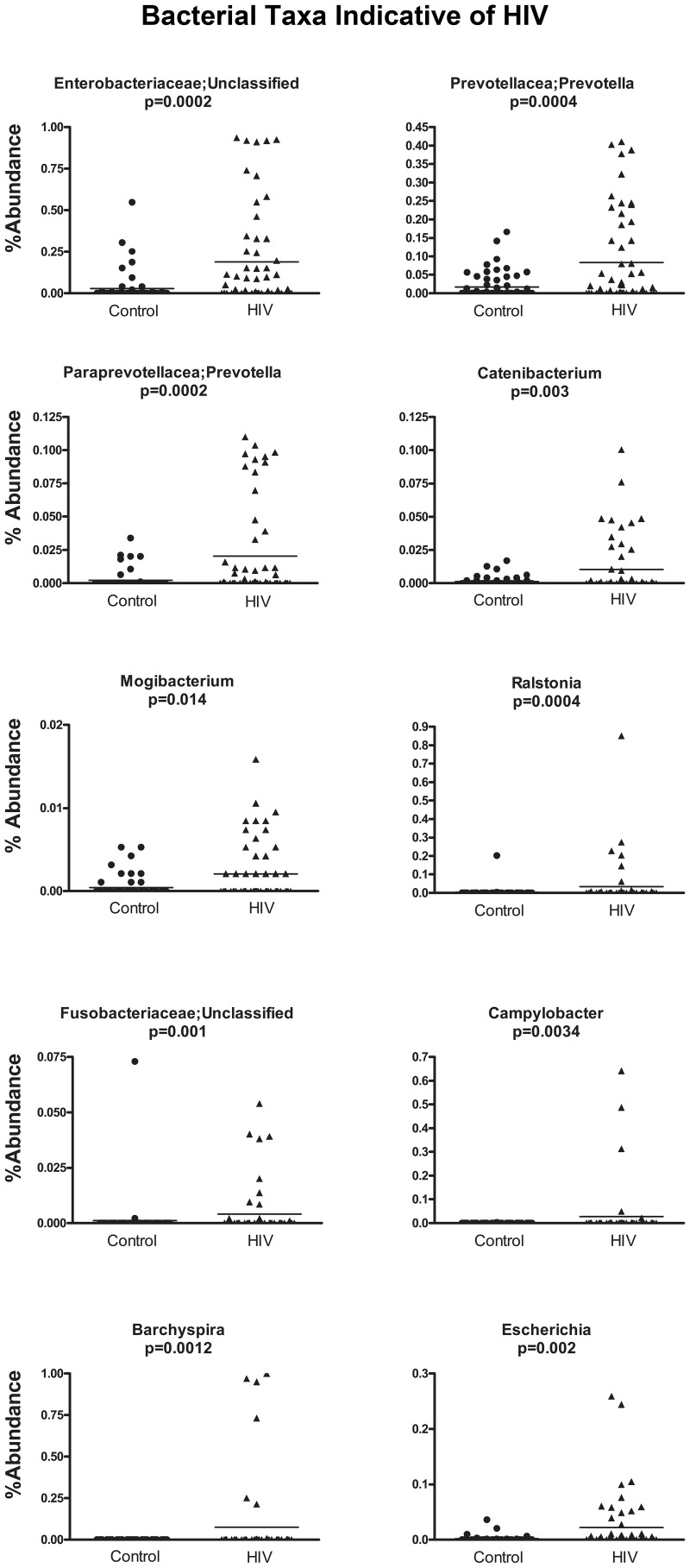
Scatterplots of bacterial taxa indicative of HIV samples. Y-axis shows percent abundance in rarified sequences for each sample. Control samples are shown as black dots and HIV samples are shown as upward black triangles. Horizontal lines denote mean value in each group. P-values shown are results from indicator species analysis. Genera and those unclassified bacterial members of families that not able to be classified down to a particular genus, that also have indicator values >15 and p<0.05 are shown.

**Figure 8:**
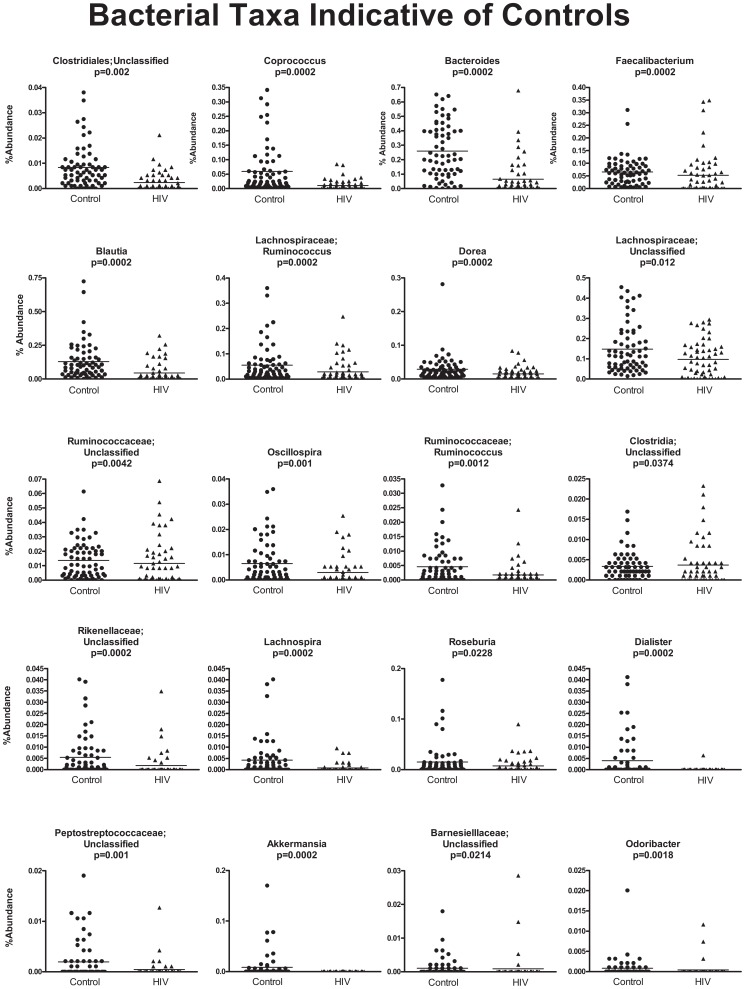
Scatterplots of bacterial taxa indicative of control samples. Y-axis shows percent abundance in rarified sequences for each sample. Control samples are shown as black dots and HIV samples are shown as upward black triangles. Horizontal lines denote mean value in each group. P-values shown are results from indicator species analysis. Genera and those unclassified bacterial members of families that not able to be classified down to a particular genus, that also have indicator values >15 and p<0.05 are shown.

In order to discover which bacterial groups are driving the differences between the HIV and the control samples, we performed an indicator species analysis at the genus level for the HIV group vs. the control cases, controlled by type of sample. Bacterial taxa that have an indicator value >15 and are significantly different (p<0.05) are shown in Figures 6–8. The magnitude of the indicator values for each taxa in HIV and controls is shown in Figure 6. HIV was associated with a significant increase in Brachyspira, Campylobacter, Catenibacterium, Escherichia and other unclassified Enterobacteriaceae, unclassified Fusobacteriaceae, Mogibacterium, Prevotella, and Ralstonia (Figure 7, p<0.05 all). On the other hand, the healthy control group had more Akkermansia, Bacteroides, Blautia, Coprococcus, Dialister, Dorea, Faecalibacterium, Lachnospira, Roseburia, Ruminococcus, Odoribacter, Oscillospira genera, as well as unclassified bacteria from the following families, Barnesiellaceae, Lachnospiraceae, Peptostreptococcaceae, Rikenellaceae, and Ruminococcaceae (Figure 8, p<0.05 all).

### Relationship between bacterial taxa in HIV-infected subject samples with serum soluble markers of immune activation

**Figure 9:**
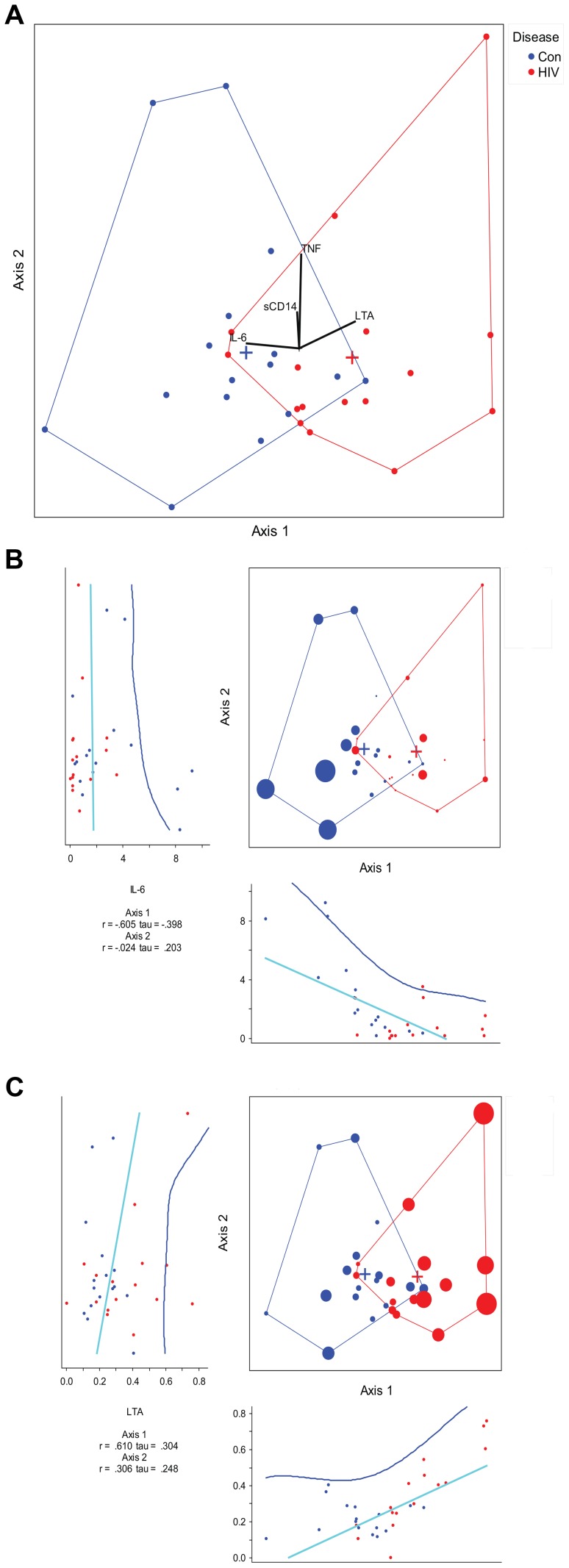
Canonical correspondence analysis of genera with measured cytokines. HIV samples are shown in red vs. control samples are shown in blue. (a)The eigenvalues for axis 1 and axis 2 are 0.107 and 0.087, respectively. The axes 1 and 2 explain 2.8% and 2.2% of the total variance, respectively. The first canonical axis is statistically significant with p = 0.003 using a randomization test where p = proportion of randomized runs with eigenvalue greater than or equal to the observed eigenvalue with 998 randomizations. The vectors in the mid portion of the graph represent cytokines or microbial translocation products. LTA increases going toward the HIV group, and IL-6 increases going toward the control group. The effect of TNF and sCD14 are minimal along the first axis of separation between the cases. (b) Canonical correspondence analysis of genera with IL-6 effect overlay. The size of the case dots correspond to the impact of IL-6 on the analysis. The regression plot for each axis coordinates and IL-6 is given below the axis for axis 1 and to the left of the axis for axis 2. (c) Canonical correspondence analysis of genera with LTA effect overlay. The size of the case dots correspond to the impact of LTA on the analysis. The regression plot for each axis coordinates and LTA is given below the axis for axis 1 and to the left of the axis for axis 2.

Soluble markers of immune activation (serum cytokines and microbial translocation markers) were measured from all subjects except five (3 subjects in the HIV-infected and 2 subjects in the control group) in whom samples were not available. We performed a canonical correspondence analysis (CCA) to look for an association between bacterial composition and serum interleukin-6 (IL-6), tumor necrosis factor alpha (TNF), lipoteichoic acid (LTA) and soluble CD14 (sCD14). CCA correlations between the cytokines (IL-6 and TNF) and microbial translocation products (LTA and sCD14) are given in Table S8. The presence of IL-6 and LTA were associated with a continuum of bacterial composition shifts from the healthy state towards HIV (shown along the first axis of the CCA) (Figure 9). The impacts of each of the cytokine and microbial translocation markers on the CCA are shown in Figures 9b–c. Increasing IL-6 was associated with controls whereas increasing LTA was associated with HIV. However, these cytokine levels failed to explain majority of the global composition changes between HIV and controls (spread across the first CCA axis), considering that the amount of cumulative variation explained was 5% of the total variance in the data. Bacterial composition-cytokine correlations were also not significant using a Monte-Carlo randomization test suggesting a weak association between these cytokines and microbial translocation products and global bacterial composition in the cases.

In situations when a handful of bacterial taxa may be driving the synthesis of these cytokines, it is certainly plausible that the individual bacterial taxa rather than the total microbial composition may be associated with cytokine levels or microbial translocation products. We tested for this by looking at Spearman's correlations between the individual bacterial taxa that were identified in the indicator species analysis and the cytokines and microbial translocation products in the HIV group. Significant potential associations with bacterial taxa in the HIV group are given in Table 3. Notably, some of these associations did not appear linear, suggesting a complex interrelationship with bacterial composition and cytokine secretion and microbial translocation products (data not shown).

**Table 3:** Statistically significant cytokine and microbial translocation product correlations with individual bacterial taxa in the HIV group.

Bacterial Taxa		IL-6		TNF		LTA		sCD14	
	Site	Spearman's rho	*p-value	Spearman's rho	*p-value	Spearman's rho	*p-value	Spearman's rho	*p-value
**Bacteroides**	Ileum	−0.836	0.019						
	LC					0.601	0.051		
**Blautia**	Fecal			0.528	0.035				
**Unclassified Clostridia**	RC			0.619	0.024				
**Unclassified Lachnospiraceae**	RC			0.575	0.040				
	Fecal			0.585	0.017				
**Fecalibacterium**	RC							−0.616	0.025
**Ruminococcus**	LC					0.799	0.003		
	Fecal			0.671	0.004				

*Unadjusted P-values.

### Relationship of the microbiome in HIV with CD4 count and viral load and other clinical characteristics

a **Clinical characteristics.** We examined the possibility of any antibiotic exposures in the HIV patients or controls affecting our results and conclusions: There were four subjects who had antibiotic exposures in the HIV group within 1 month of sample collection. In total, 2/22 of the control subjects and 8/21 of the HIV subjects had a history of antibiotic use anytime within the past 1 year. The two control subjects had not used any antibiotics within the last 3 months, but were exposed to antibiotics within 12 months. Amongst the HIV subjects, one was on TMP-SMZ prophylaxis and another was prescribed this although did not report himself as taking it. In the other 6 HIV-positive subjects, the last antibiotic use in the subjects were three weeks prior to sample collection (n = 2), 4 months prior to sample collection (n = 1), 6 months prior to sample collection (n = 1), 7 months prior to sample collection (n = 1), and 12 months prior to sample collection (n = 1). When samples from subjects who have any antibiotic exposures were examined against the rest of the samples in terms of diversity indices, we noted no differences for samples collected from subjects with an antibiotic exposure within the past 1 month versus those with no antibiotic exposures (Figure S7). Similarly, we noted no difference between the samples collected from subjects with an antibiotic exposure within the past 3 months versus those with no antibiotic exposures (Figure S7). However, there appeared to be some difference between samples collected from subjects with an antibiotic exposure within the past 1 year versus those with no antibiotic exposures (Figure S7).

We then eliminated all the samples with any antibiotic exposures from the diversity analyses, and reanalyzed the data. The reduction in diversity in the HIV samples versus controls was preserved when all samples were examined (Figure S8). By site, the reduction in diversity in the HIV samples versus controls was preserved in the TI and RC, whereas differences in the LC or F samples were not apparent (Figure S8). In subject group comparisons, in a NMDS analysis using Bray-Curtis distances and in a PCO plot using Unifrac distances, we noted no differences between samples collected from subjects with antibiotic exposure within 1 month against those collected from subjects with no antibiotic exposures (Figures S9). We also noted no difference between the samples collected from subjects without antibiotic exposures and those with antibiotic exposures within 3 months or within 1 year of mucosal sampling (Figure S9). Therefore, it is highly unlikely that antibiotic use in the HIV setting explains the microbiome changes that have been noted above.Among the HIV subjects, only four subjects admitted to any drug use and only three of these were current users. The drugs used were cocaine (n = 3), marijuana (n = 1), and methamphetamines (n = 1). When samples from these subjects were compared to the samples from non-drug users, we noted no differences in NMDS or PCO plots (Figure S10).

b **CD4+ T- cell counts.** Diversity indices did not show a difference between those HIV infected subjects with high and low CD4 counts at cutoffs of 200 and 500 in all subjects as well as those subjects without any antibiotic exposures (Figures S11 and S12). In PCO analysis using Unifrac distances and in NMDS analysis using Bray-Curtis distances, samples belonging to subjects whose CD4+ T cell counts were <200 were not appearing clustered together, and were not separated from samples belonging to subjects whose CD4+ T cell counts were >200. (Figure S13). No apparent separation was seen for samples coming from subjects whose CD4+ T cell counts were <500, versus those with CD4+ T cell counts >500 (Figure S13). There were no significant correlations between CD4+ T cell count and individual bacterial taxa.
c **Viral load.** In our dataset, seventeen out of 21 HIV- infected subjects had undetectable viral loads, so it was not possible to perform a correlation between the microbiome and viral load. In alpha diversity or ordination analyses, we did not note a significant difference between samples from viremic subjects versus non-viremic subjects (Figures S14 and S15).

## Discussion

This study represents a first global look at the lower GI tract microbiome in HIV-infected subjects using sequencing technologies. Our data shows that the lower intestinal mucosal bacterial populations in HIV-infected subjects are less diverse, definitely distinct from non-HIV controls, and composed more frequently of bacterial populations that are potentially pathogenic. Such disarray in the lower gut microbiome during HIV-infection goes along with findings of increased gut permeability in HIV and may be one of the contributing etiologic factors to HIV progression, warranting further investigation. Notably, the microbiome in HIV-infected subjects is so different that the cases can be distinctly separated from healthy controls based on global microbiome composition. In many other pyrosequencing experiments in human subjects, in unconstrained ordination analyses, the variability of the GI tract microbiome itself between subjects or samples, usually outweighs the effects seen as a result of disease. Such a large magnitude of disarray in the GI tract microbiome in HIV signals the importance of the GI tract microbiome in HIV infection, and could be both a consequence of HIV infection itself and/or a contributor to disease progression.

When the individual genera associated with HIV-infected subjects and controls are examined, an important finding is the loss of a significant set of genera associated with controls in the HIV cases, typical of a dysbiotic microbiome. In many instances these are genera that are thought to be beneficial such as Bacteroides, which was negatively associated with IL-6 in our HIV cases. The network analysis also suggests that HIV is characterized by the loss of OTUs, and a change across all samples. In fact, while the HIV microbiome is a less diverse microbiome, the samples in the HIV group are farther apart from each other in our network analysis suggesting that the gain of bacteria among HIV samples is rather numerous and can vary across persons infected with HIV. Furthermore, nearly all of the bacterial taxa we have found to be associated with HIV in our study have also shown to be potentially pathogenic organisms in many other disease states, though some are rarely pathogenic. When we look at these one by one, the following can be said about the genera associated with HIV: Escherichia and other genera from Enterobacteriaceae, Campylobacter, various genera from Fusobacteriaceae are common pathogens of UTI, bacteremia, gastrointestinal infections, and periodontitis and necrotizing infections/abscesses. Catenibacterium has previously been associated with presence of uremia in subjects with end stage renal disease [13]. Mogibacterium has been found in the sputum of people with tuberculosis [14] and has also been associated with periodontitis [15], [16] and endodontic infections [17]. Ralstonia are plant pathogens but have been emerging in case reports of community acquired pneumonia [18], [19] as well as becoming increasingly identified as infectious agents in cystic fibrosis patients [20],immunocompromised individuals with hematological malignancies [21], [22], and in nosocomial infections [23]. Brachyspira is the cause of intestinal spirochetosis in both animals and humans; and have previously been described as a cause of chronic diarrhea in HIV subjects [24]. While Prevotella are commonly found in healthy individuals in the GI tract, they could be pathogenic at other sites across the body such as the oral mucosa or the vagina. Prevotella are associated with anaerobic infections of the respiratory tract such as aspiration pneumonia, lung abscess, chronic otitis media and sinusitis; abscesses round the mouth; urinary tract infections; brain abscesses; osteomyelitis ; periodontal disease; in addition to their well-documented presence in bacterial vaginosis.

Previous studies on the vaginal microbiome in HIV also have suggested a significant change at this site [25]–[29], with an increased diversity in the vaginal environment in HIV [30]. In contrast, our results suggest a decreased diversity in the GI tract microbiome of HIV-infected subjects. This may be due to the inherent nature of the bacterial communities at these different mucosal sites: There is low diversity typically seen in the vaginal environment with high specialization for certain functions, such as regulation of vaginal pH, which shows correlation with microbiome composition [31]. In contrast, high diversity is typically seen in the GI tract in the healthy state [31], with reductions in diversity seen in most disease states investigated to date across multiple studies (e.g. inflammatory bowel disease and colon cancer) [32]–[34]. One of the bacterial taxa associated with bacterial vaginosis in the vaginal environment in women with and without HIV has been Prevotella [30], which was also identified to be increased in the GI tract specimens from HIV cases in our study. This co-occurrence of Prevotella in the HIV GI tract specimens and in bacterial vaginosis may suggest that the gut could be one source of colonization of the vagina with Prevotella in bacterial vaginosis, which in turn is an important risk factor for heterosexual HIV transmission. Therefore, we suspect that the microbiome changes in HIV across the body could be wide-spread and could potentially be related to each other. Further studies that concomitantly examine the GI tract and the vaginal microbiome are needed to increase our understanding of the role of the GI tract microbiome in altering the vaginal environment in HIV and thereby as a risk factor for HIV transmission.

On the contrary to the human observations in our study, two separate studies on SIV-infected macaques have not shown an alteration in gut bacterial composition [3], [9]. This significant discrepancy between our findings in HIV-infected humans and those in SIV-infected macaques could relate to a number of factors: There could be intrinsic differences between SIV as model of disease versus HIV ; the presence of colitis in some SIV-infected macaques may have affected or surpassed the magnitude of the change in microbiome composition by SIV itself making these changes difficult to detect; and/or the discrepancy could be explained by the study of fecal samples in the case of SIV-infected macaques in the two previously published studies, versus the study of colonic tissue biopsies in the case of HIV-infected humans in our study. Alternatively, it is also possible that the major driver of bacterial compositional alterations in HIV-infected humans could be disease related factors such as the presence of HAART or other treatments that may have been administered to humans over the course of disease. Alternatively, lifestyle factors (that are tightly controlled in animal models) rather than the presence of HIV infection could have also resulted in changes in the bacterial microbiome in HIV-infected humans. Future studies should carefully characterize such potential confounders in humans such as diet, treatment duration, as well as antibiotic exposures in a longitudinal manner.

Compared to other studies that have profiled individual bacterial orders with PCR in the GI tract in HIV in humans, our study is confirmatory: Previous studies have shown increases in Enterobacteriales and Bacteroidales orders in HIV [6], [35] corresponding to the rises in Escherichia and other genera from Enterobacteriaceae and rises in Prevotella (at the expense of other Bacteroidales such as Bacteroides) in our study, respectively. We have also identified a number of other genera that have not been associated with HIV before. During the revision of this manuscript, an additional study examining the bacterial microbiome with a microarray technique in rectal biopsies of 25 HIV-infected humans has been published [36]. Findings in this study are largely parallel to ours: There is an increase in pathogenic Proteobacteria and a decrease of the Bacteroidia members in the dysbiotic mucosal-adherent bacterial community in the rectum. Similar to our study, there was not a strong association between peripheral blood CD4+ T cell counts and microbiome composition. Our study adds to these published findings in several ways: The dysbiosis in HIV occurs in multiple locations throughout the lower GI tract. We also note a reduction in the diversity of the microbiome which was most evident on the mucosal surface (especially the right colon) and less so in fecal samples. We note a disarray and heterogeneity in microbiome composition in the HIV group resulting in less connectedness of the microbiome in our network analysis, which in turn may affect the ability of the microbiome to suppress or constrain pathologic organisms in the gut and increase the susceptibility to further perturbations. Hence, our findings in conjunction with other studies suggest that there may be a potentially pathogenic but typically commensal microbiome pattern in HIV that could be modulated for therapeutic gains. Future larger scale studies are needed to confirm HIV specific microbiome patterns in the GI tract.

One possible mechanism by which the GI tract microbiome could modulate HIV progression and related disease states, could be through immune alterations resulting in the release of inflammatory cytokines that occur as a result of gut barrier dysfunction, which has been well described in HIV [7], [37]–[40]. Our study found associations with inflammatory cytokines in the blood and the microbiome. However, it appears that the effect of the serum cytokines and microbial translocation products tested is a weak one and may perhaps require larger numbers of subjects to study. Perhaps circulating cytokines or other immunological correlates of HIV infection other than the ones examined in this study (such as the recently identified increased kynurenine production [36]) or cytokine/chemokine levels in the gut mucosa, or additional mechanisms that result in soluble factors or direct cell to cell contact between bacteria and the intestinal mucosal surface have a larger magnitude of association with the potentially pathogenic GI tract microbiome in HIV, and now require further study.

The strength of our study is the use of state of the art pyrosequencing technologies to evaluate the lower intestinal microbiome; the use of mucosal samples throughout the lower GI tract for pyrosequencing in addition to fecal samples; and multiple approaches to data analysis to gain insights into the data. We believe mucosal biopsy samples may give a better understanding of the interactions between the microbiome and disease states due to the close proximity of epithelial cells with bacteria. While the microbiome in stool samples is thought to grossly mirror the microbiome on the mucosal surface in the healthy state, not all members of the fecal community are members of the mucosal community; and fecal microbiome composition is also subject to change with transient members that may come and go, as part of food/other ingested materials [41], [42]. The fecal microbiome is also generally more diverse than the mucosal microbiome. This increased level of microbial diversity could make subtle changes in the disease setting much harder to detect or less apparent in fecal samples. This may also be one reason to note a greater difference in the mucosal samples versus the fecal samples in our study. Our study is limited by its cross-sectional design and it is hypothesis generating; but future longitudinal studies are needed to infer cause-effect relationships between HIV transmission, spread and progression and the GI tract microbiome. Another limitation of our study is the fact that the patients in our study were on HAART. It is currently unknown whether the therapy itself could lead to changes in the GI tract microbiome. In the future, bacterial composition in the lower GI tract will also have to be explored in patients who are not on any medications and the changes seen in this study will have to be confirmed in untreated HIV populations.

Strategies modulating the GI tract microbiome have been underway as part of the efforts to devise therapeutic strategies directed against harmful immune activation in HIV. One such strategy could be dietary manipulation or manipulation of the microbiome with prebiotics and/or probiotics [8], [43]–[45]. Our findings add to the body of literature on this topic and also suggest that there may be a therapeutic potential for such interventions. Future studies exploring the diets of HIV patients in conjunction with disease stage and the GI tract microbiome are needed to explore the role of dietary manipulation, prebiotics and probiotics as part of HIV treatment.

In conclusion, there are changes in the lower GI tract microbiome in HIV characterized by loss of various commensal bacterial genera and gain of potentially pathogenic ones, and this finding now paves the way to future studies that examine further relationships between the GI microbiome and HIV transmission as well as progression.

## Materials and Methods

### Subjects and samples

Colonic biopsy samples and fecal samples were obtained from the tissue bank at Rush University Medical Center, Division of Gastroenterology, Hepatology and Nutrition, from 21 subjects with HIV and 22 control subjects, after obtaining study approval from the Rush IRB. All subjects gave written and verbal informed consent prior to tissue collection under the tissue bank IRB at Rush University and were recruited from the same geographic area and one single outpatient endoscopy lab. The utilized tissue bank obtained samples from the terminal ileum, right colon, left colon and luminal fecal samples at the time of the subject's colonoscopy with a standard 2.2 mm biopsy forceps or a luken's trap, respectively. All samples were placed in a cryovial and flash frozen in liquid nitrogen in the endoscopy room and stored in −80 C freezers. Samples were analyzed anonymously under a research protocol which was also approved by the Rush University Medical Center IRB. The characteristics of the human subjects and the collected tissues are given in Tables 1, S1 and S2. All subjects received a polyethylene glycol (PEG) based bowel prep within 24 hours of colonoscopy, except one subject who received a sodium phosphate based bowel prep. There were no differences in samples from subjects exposed to various PEG based preps in a PCO analysis using Unifrac (Figure S16).

### Sequencing

DNA was extracted using a commercially available kit, FastDNA Spin Kit for Soil, (MP Biomedicals, Solon, OH 44139 USA), using the manufacturer's recommended protocol. The adequacy of the amount of extracted DNA from samples was verified with fluorometric quantitation (Qubit, Life Technologies, Grand Island, NY 14072) and samples with inadequate amounts of template DNA were not sequenced. 5′GAGTTTGATCNTGGCTCAG3′ forward primer and 5′GNTTTACNGCGGCKGCTG3′ reverse primers were used to pyrosequence the 16S rDNA on a 454 GS FLX platform, with barcoding, using titanium kits [46].

### Sequence processing and quality assessment

Custom C# and python scripts as well as python scripts in the Quantitative Insights Into Microbial Ecology (QIIME) software pipeline (VirtualBox Versions 1.5 and 1.6) were used to process the sequencing files [47]–[50]. Two sequence runs were performed with about equal distribution of HIV and control cases in both runs. The sequence outputs were filtered for low quality sequences (defined as any sequences that are <200 bps or >1000 bps, sequences with any nucleotide mismatches to either the barcode or primer, sequences with homopolymer runs >6, sequences with an average quality score of <25, sequences with ambigious bases >6) and were truncated at the reverse primer. Sequences were denoised using USEARCH [51], and chimera checked with UCHIME [52] and Chimera Slayer [53]. Operational taxonomic units (OTUs) were picked using uclust [51]at 97% similarity, and representative sequences were generated. Sequences were aligned with PyNAST [54] and taxonomy assignment was performed in Qiime 1.6VB against the Qiime 1.6 version of Greengenes database [55], [56] using the RDP classifier [57] at a 80% bootstrap value threshold. An approximately-maximum-likelihood phylogenetic tree was created using FastTree v2.1.3 [58]. Differential dispersion of the samples by runs for batch effects were also checked usingPCO using Unifrac distances. While run related batch effects were seen, these failed to explain the group differences observed between HIV and controls (Figure S17).

### Lab measurements

Serum specimens were evaluated in duplicate batches for circulating levels of IL-6, and TNF-α using the Human High Sensitivity Cytokine/Chemokine kit (EMD Millipore, Billerica, MA) according to manufacturer recommended protocols. All kits were read using a Luminex 100 IS System (Luminex Corp, Austin, TX) by the Rush Proteomics and Biomarkers Core Facility with biomarker concentrations calculated using a 5-parametric curve fit using xPonent 3.2 software (Luminex Corp). Median %CV and assay recovery values all fell within acceptable limits specified by EMD-Millipore. The serum levels of soluble CD14 (sCD14) and Lipoteichoic acid (LTA) was determined by ELISA.

### Statistics

Qiime 1.5 was used calculate Bray-Curtis similarity and Unifrac distances based on OTUs, and NMDS and PCO coordinates, Unifrac tests; UPGMA clustering; and calculate the bipartite network attributes. UPGMA dendogram and phylogenetic trees were visualized in FigTree v1.3.1. Cytoscape v2.8.1 was used to visualize the network; and calculate network and node statistics. PerMANOVA, Indicator species analysis (ISA) [59], CCA and were performed in PC-ORD. For PerMANOVA analyses, we used the Bray-Curtis similarity at the genus level on rarified and log transformed data. In order to adjust for unequal sample sizes in groups, we randomly subsampled the groups 1000 times, stratified by sample site (I,RC,LC, and F), and performed a randomization test of significance of pseudo F values, with 4999 randomizations for each model, and collated the results from the 1000 subsamplings. For ISA analyses, we used rarified and log transformed data and the analysis was blocked for sample type. For CCA analyses, in order to adjust for unequal sample sizes in groups, we subsampled 8 cases from each site for each group randomly. Axis scores were standardized and centered to unit variance. Scores for graphing samples were linear combinations of genera. We performed a randomization test of the eigenvalues of the canonical axes with 1000 randomizations. We also performed a randomization test with 1000 randomizations for as species-environment correlations for which the null hypothesis was that there is no structure in the bacterial genera and therefore no relationship between the bacterial genera and the cytokine matrices. SPSS (Version 17.0.0, Chicago, IL, USA) was used to analyze clinical metadata and to correlate bacterial taxa (which were found to be significant in the indicator species analysis) to lab measurements in the HIV group. In SPSS, T- tests or ANOVA were used to analyze differences for parametric data satisfying test assumptions; Kruskal- Wallis or Mann-Whitney tests or median tests were used to analyze nonparametric data; Chi-Square or t–tests were used to detect differences in proportions between groups, as appropriate. In SPSS, bacterial taxa count data was log transformed and most taxa were observed to be non-normally distributed. Spearman's correlations were calculated. Correlations that were based mostly on zeros or few sequences were not reported as significant. GraphPadPrism was used to generate scatterplots of bacterial taxa.

## Supporting Information

Figure S1 Jackknifed principal coordinates analysis of all of the samples using Unifrac. HIV samples are shown in blue vs. control samples are shown in red. The jackknifed estimates are represented by the confidence ellipsoids around each sample point, representing the degree of variation from one replicate to the next.
(TIF)

Figure S2 Jackknifed principal coordinates analysis of all of ileal samples using Unifrac. The jackknifed estimates are represented by the confidence ellipsoids around each sample point, representing the degree of variation from one replicate to the next.
(TIF)

Figure S3 Jackknifed principal coordinates analysis of all of right colon samples using Unifrac. The jackknifed estimates are represented by the confidence ellipsoids around each sample point, representing the degree of variation from one replicate to the next.
(TIF)

Figure S4 Jackknifed principal coordinates analysis of all of left colon samples using Unifrac. The jackknifed estimates are represented by the confidence ellipsoids around each sample point, representing the degree of variation from one replicate to the next.
(TIF)

Figure S5 Jackknifed principal coordinates analysis of all of fecal samples using Unifrac. The jackknifed estimates are represented by the confidence ellipsoids around each sample point, representing the degree of variation from one replicate to the next.
(TIF)

Figure S6 Network diagram of the bipartite OTU and sample network. HIV sample nodes are colored in red, control sample nodes are colored in blue, and OTU nodes are colored as small white dots. Edges originating from HIV sample nodes are colored in pink, and edges originating from control nodes are colored in lavender. Samples that are closer together in the network diagram share more bacterial OTUs than others, and are closer neighbors.
(TIF)

Figure S7 Alpha diversity assessed by diversity indices in samples with antibiotic exposures versus those with no antibiotic exposures. Samples collected from subjects with antibiotic exposures are shown in blue and samples collected from subjects without antibiotic exposures are shown in red. Diversity indices shown are OTU richness (panels (a–c)), Chao1 index (panels (d–f)), and Phylogenetic Diversity (PD) Whole Tree metric (panels (g–i)). Samples collected from subjects within 1 month of antibiotic exposure are shown in panels (a),(d),(g); samples collected from subjects within 3 months of antibiotic exposure are shown in panels (b),(e),(h) ; samples collected from subjects within 1 year of antibiotic exposure are shown in panels (c),(f),(i).
(TIF)

Figure S8 Alpha diversity assessed by diversity indices in samples without any antibiotic exposures. HIV cases versus controls are shown by sample site. HIV cases have decreased diversity compared to controls. Diversity indices shown are OTU richness (panels (a–e)), Chao1 index (panels (f–j)), and Phylogenetic Diversity (PD) Whole Tree metric (panels (k–o)). All samples are shown in panels (a),(f),(k);and HIV samples are in blue; Control samples are in red in these panels. Samples from ileum are shown in panels (b),(g),(l); and HIV ileal samples are in red; Control ileal samples are in purple in these panels. Samples from right colon are shown in panels (c),(h),(m); and HIV right colon samples are in orange; Control right colon samples are in light blue in these panels. Samples from left colon are shown in panels (d),(i),(n); and HIV left colon samples are in green; Control left colon samples are in pink in these panels. Fecal samples are shown in panels (e),(j),(o); and HIV fecal samples are in blue; Control fecal samples are in yellow in these panels.
(TIF)

Figure S9 Beta diversity assessed by NMDS and PCO in samples with antibiotic exposures versus those with no antibiotic exposures. Samples collected from subjects with antibiotic exposures are shown in blue and samples collected from subjects without antibiotic exposures are shown in red. For NMDS the Bray-Curtis similarity at the OTU level was used. For PCO, Unifrac distances were used. Panels (a–c) show the results for NMDS, and panels (d–f) show the results for Unifrac. Panels (a) and (d) show samples collected from subjects within 1 month of antibiotic exposure in blue; Panels (b) and (e) show samples collected from subjects within 3 months of antibiotic exposure in blue; Panels (c) and (f) show samples collected from subjects within 1 year of antibiotic exposure in blue. No differences are noted in beta diversity.
(TIF)

Figure S10 Beta diversity assessed by NMDS and PCO in samples from illicit drug users versus non-users. For NMDS (panel (a)), the Bray-Curtis similarity at the OTU level was used. For PCO (panel (b)), Unifrac distances were used. Samples from non-users are in blue; samples from current users are in red (n = 11); samples from former users are in orange (n = 4); samples for which no data is available are in purple (n = 2). No differences are noted in beta diversity.
(TIF)

Figure S11 Alpha diversity assessed by diversity indices in HIV samples with CD4+ T cell counts less than 200 versus those with CD4+ T cell counts more than 200. Samples from cases with counts <200 are in orange; samples from cases with counts >200 are in blue. Panels (a–c) show results of diversity indices in all samples and panels (d–f) show results of diversity indices in samples collected from subjects without any antibiotic exposures within 1 year of mucosal sampling. Diversity indices shown are OTU richness (panels (a–d)), Chao1 index (panels (b–e)), and Phylogenetic Diversity (PD) Whole Tree metric (panels (c–f)). No differences are noted.
(TIF)

Figure S12 Alpha diversity assessed by diversity indices in HIV samples with CD4+ T cell counts less than 500 versus those with CD4+ T cell counts more than 500. Samples from cases with counts <500 are in orange; samples from cases with counts >500 are in blue. Panels (a–c) show results of diversity indices in all samples and panels (d–f) show results of diversity indices in samples collected from subjects without any antibiotic exposures within 1 year of mucosal sampling. Diversity indices shown are OTU richness (panels (a–d)), Chao1 index (panels (b–e)), and Phylogenetic Diversity (PD) Whole Tree metric (panels (c–f)). No differences are noted.
(TIF)

Figure S13 Beta diversity assessed by NMDS and PCO in samples with CD varying CD4+ T cell count thresholds. In panels (a) and (c), samples from cases with counts <200 are in orange; samples from cases with counts >200 are in blue. In panels (b) and (d), samples from cases with counts <500 are in orange; samples from cases with counts >500 are in blue. For NMDS shown in panels (a) and (b), the Bray-Curtis similarity at the OTU level was used. For PCO shown in panels (c) and (d), Unifrac distances were used. No differences are noted in beta diversity.
(TIF)

Figure S14 Alpha diversity assessed by diversity indices in HIV samples with detectable viremia versus those with viral suppression. Samples from controls are in blue; samples from HIV infected subjects with viremia are in red; and samples from HIV infected subjects who have viral suppression are in orange. Diversity indices shown are OTU richness (panel (a)), Chao1 index (panel (b)), and Phylogenetic Diversity (PD) Whole Tree metric (panel (c)). No differences are noted.
(TIF)

Figure S15 Beta diversity assessed by NMDS and PCO in samples from HIV infected subjects with and without viremia. Samples from HIV infected subjects with viremia are in red; and samples from HIV infected subjects who have viral suppression are in orange. For NMDS shown in panel (a), the Bray-Curtis similarity at the OTU level was used. For PCO shown in panels (b), Unifrac distances were used. No differences are noted in beta diversity.
(TIF)

Figure S16 Beta diversity assessed by PCO using Unifrac distances for samples in different runs. In panel (a), samples in Run 1 are in red; and samples in Run 2 are in blue. In panel (b–d), control samples in Run 1 are shown in red; HIV samples in Run 1 are shown in blue, control samples in Run 2 are shown in orange, and HIV samples in Run 2 are shown in green. Panel (b) shows results for both runs. Panel (c) shows results for Run 1 by disease presence. Panel (d) shows results for Run 2 by disease presence. Separation of HIV and HC samples are seen in both runs when examined individually in panels (c) and (d). Run effects are in a vertical direction along axis 2 and disease effects are in a horizontal direction along axis 1, suggesting little to no effect of batch related variability in the results.
(TIF)

Figure S17 Beta diversity assessed by PCO using Unifrac distances in samples with various bowel preps. Samples from subjects taking PEG+vitC (i.e. Moviprep) are in red (n = 41); samples from subjects taking PEG are in orange (n = 77); and samples from subjects taking sodium phosphate tablets are in blue (n = 3). No differences are noted in beta diversity.
(TIF)

Table S1 Demographics of HIV and control subjects.
(DOCX)

Table S2 Clinical characteristics of HIV Subjects.
(DOCX)

Table S3 Network statistics for the overall bipartite sample and OTU network.
(DOCX)

Table S4 Phyla level bacterial microbiome composition in control and HIV samples.
(DOCX)

Table S5 Class level bacterial microbiome composition in control and HIV samples.
(DOCX)

Table S6 Order level bacterial microbiome composition in control and HIV samples.
(DOCX)

Table S7 Family level bacterial microbiome composition in control and HIV samples.
(DOCX)

Table S8 Correlations between cytokines (Kendall's tau) used in CCA analysis.
(DOCX)
