# Supplementary figures and images for: A Compositional Look at the Human Gastrointestinal Microbiome and Immune Activation Parameters in HIV Infected Subjects
Source: PLoS Pathog. 2014 Feb 20;10(2):e1003829. doi: 10.1371/journal.ppat.1003829 (PMC3930561; doi:10.1371/journal.ppat.1003829)

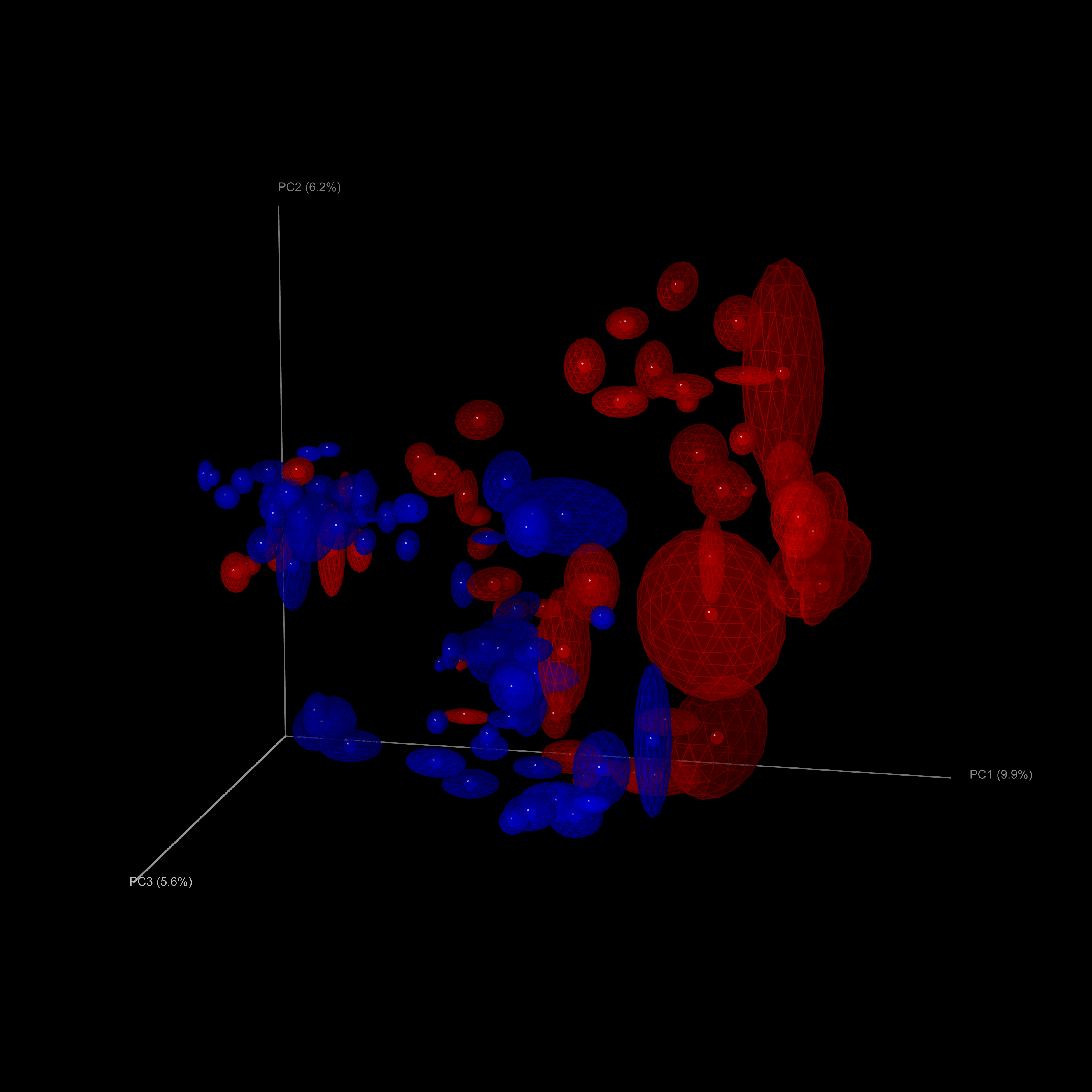

Supplement: Figure S1 — Jackknifed principal coordinates analysis of all of the samples using Unifrac. HIV samples are shown in blue vs. control samples are shown in red. The jackknifed estimates are represented by the confidence ellipsoids around each sample point, representing the degree of variation from one replicate to the next. (TIF) [file ppat.1003829.s001.tif]

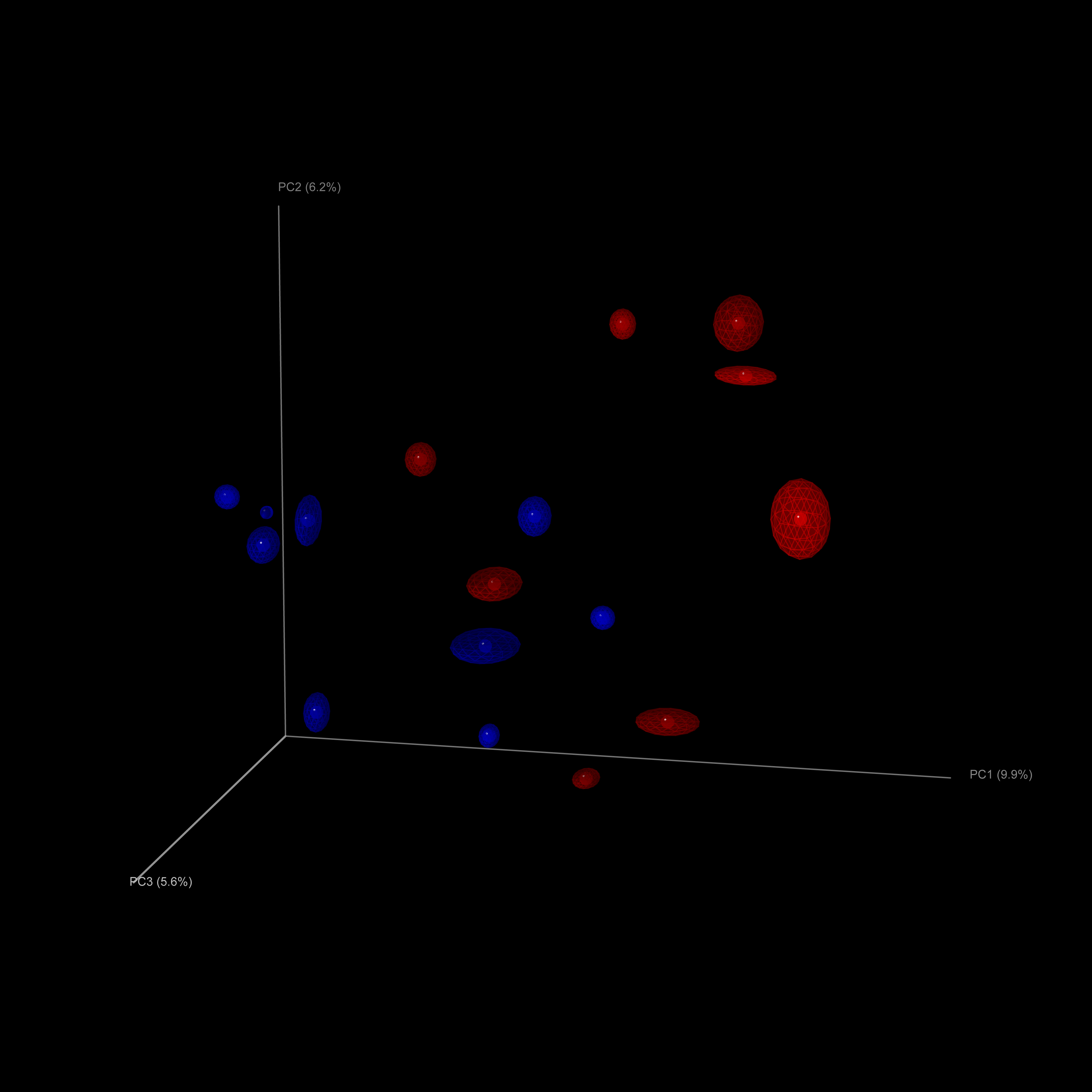

Supplement: Figure S2 — Jackknifed principal coordinates analysis of all of ileal samples using Unifrac. The jackknifed estimates are represented by the confidence ellipsoids around each sample point, representing the degree of variation from one replicate to the next. (TIF) [file ppat.1003829.s002.tif]

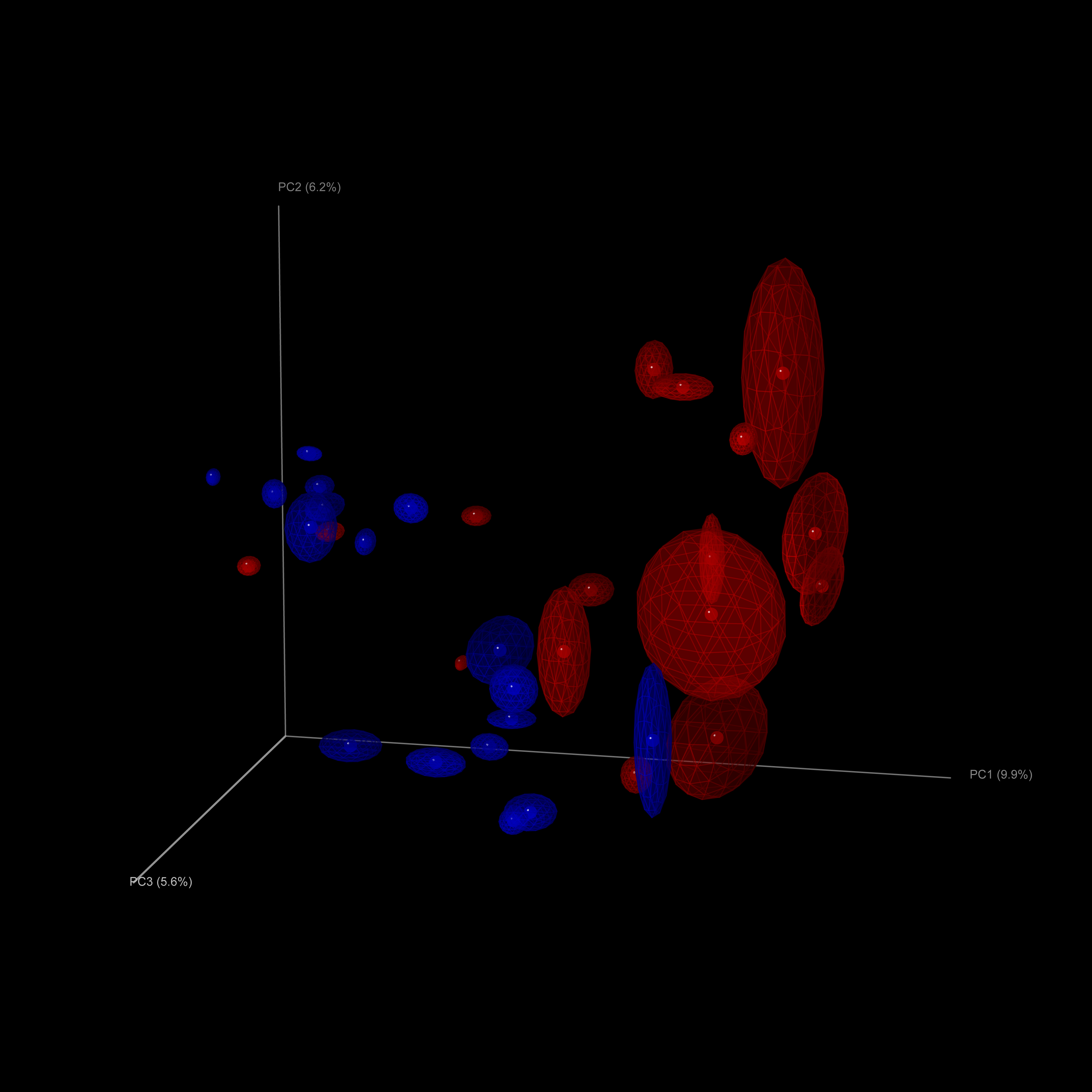

Supplement: Figure S3 — Jackknifed principal coordinates analysis of all of right colon samples using Unifrac. The jackknifed estimates are represented by the confidence ellipsoids around each sample point, representing the degree of variation from one replicate to the next. (TIF) [file ppat.1003829.s003.tif]

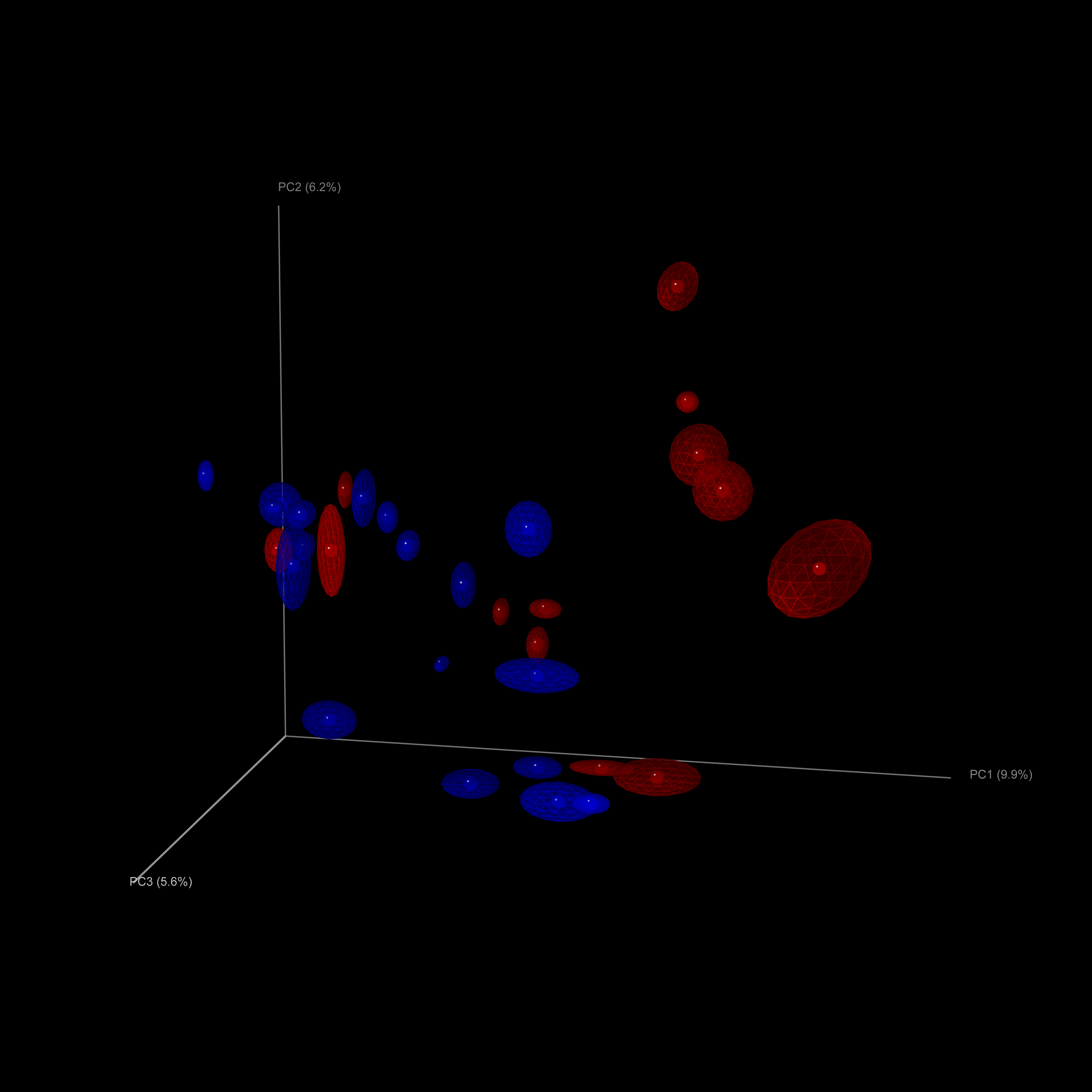

Supplement: Figure S4 — Jackknifed principal coordinates analysis of all of left colon samples using Unifrac. The jackknifed estimates are represented by the confidence ellipsoids around each sample point, representing the degree of variation from one replicate to the next. (TIF) [file ppat.1003829.s004.tif]

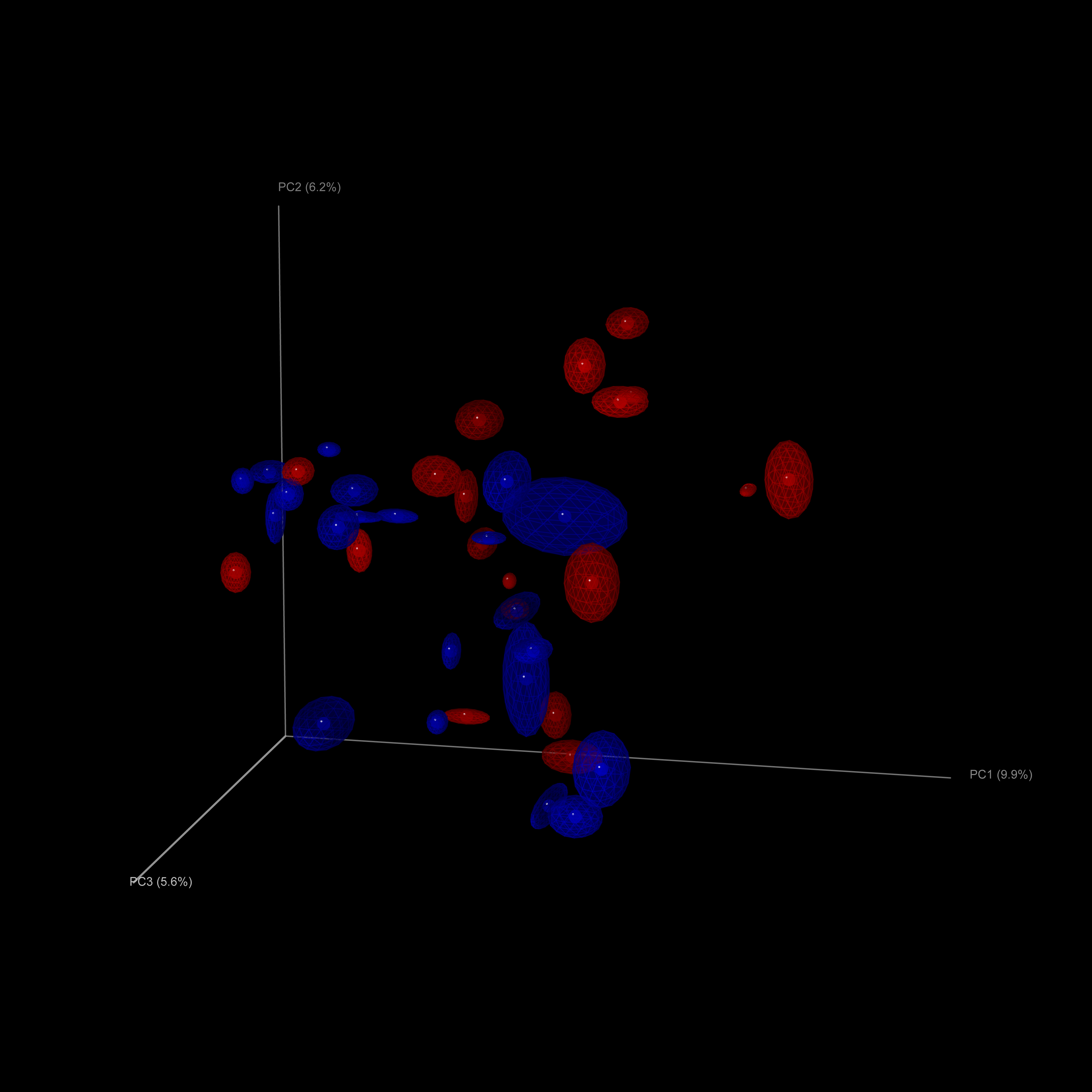

Supplement: Figure S5 — Jackknifed principal coordinates analysis of all of fecal samples using Unifrac. The jackknifed estimates are represented by the confidence ellipsoids around each sample point, representing the degree of variation from one replicate to the next. (TIF) [file ppat.1003829.s005.tif]

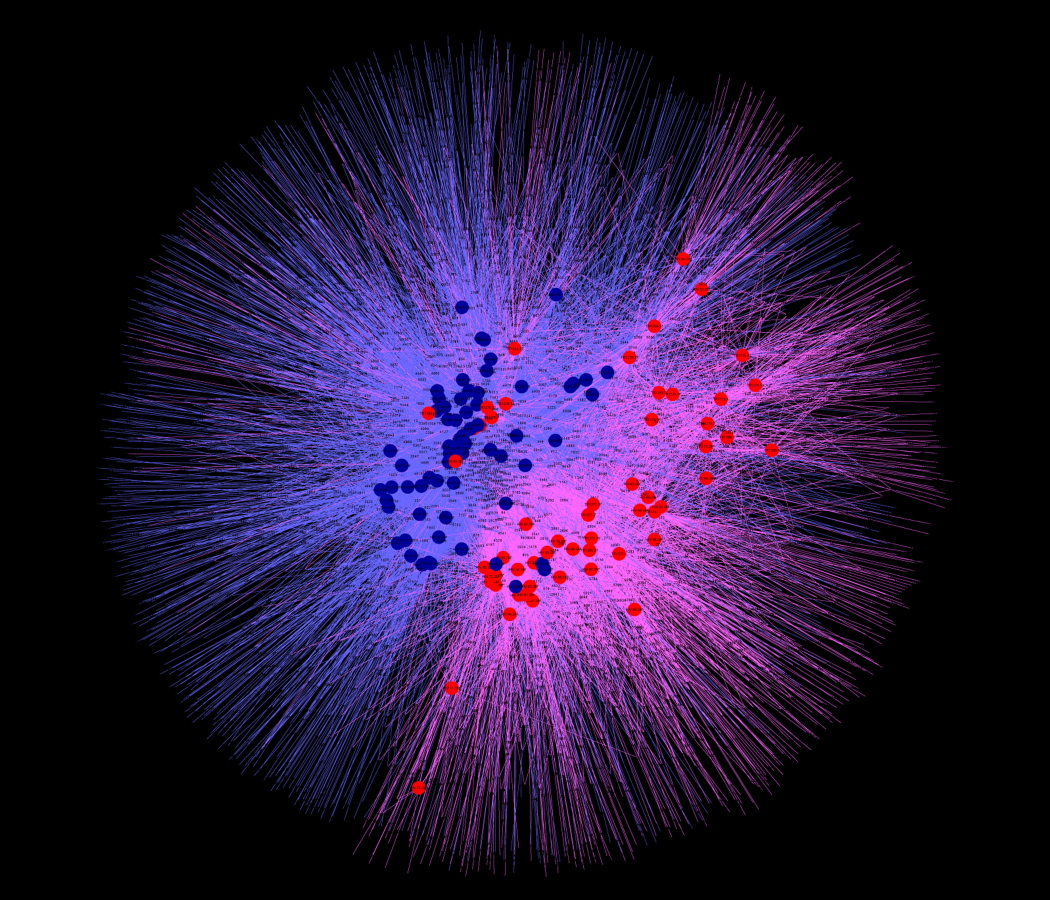

Supplement: Figure S6 — Network diagram of the bipartite OTU and sample network. HIV sample nodes are colored in red, control sample nodes are colored in blue, and OTU nodes are colored as small white dots. Edges originating from HIV sample nodes are colored in pink, and edges originating from control nodes are colored in lavender. Samples that are closer together in the network diagram share more bacterial OTUs than others, and are closer neighbors. (TIF) [file ppat.1003829.s006.tif]

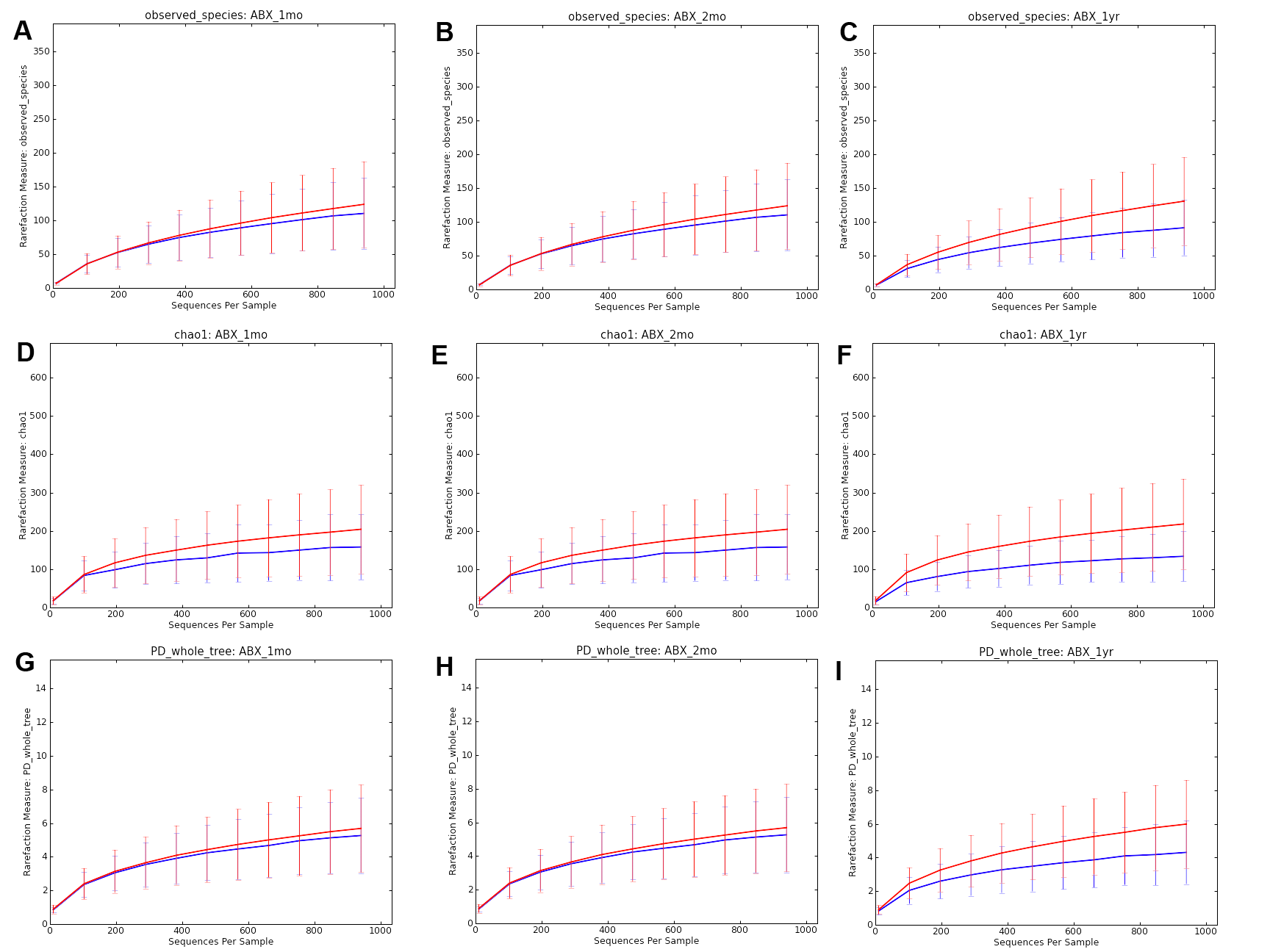

Supplement: Figure S7 — Alpha diversity assessed by diversity indices in samples with antibiotic exposures versus those with no antibiotic exposures. Samples collected from subjects with antibiotic exposures are shown in blue and samples collected from subjects without antibiotic exposures are shown in red. Diversity indices shown are OTU richness (panels (a–c)), Chao1 index (panels (d–f)), and Phylogenetic Diversity (PD) Whole Tree metric (panels (g–i)). Samples collected from subjects within 1 month of antibiotic exposure are shown in panels (a),(d),(g); samples collected from subjects within 3 months of antibiotic exposure are shown in panels (b),(e),(h) ; samples collected from subjects within 1 year of antibiotic exposure are shown in panels (c),(f),(i). (TIF) [file ppat.1003829.s007.tif]

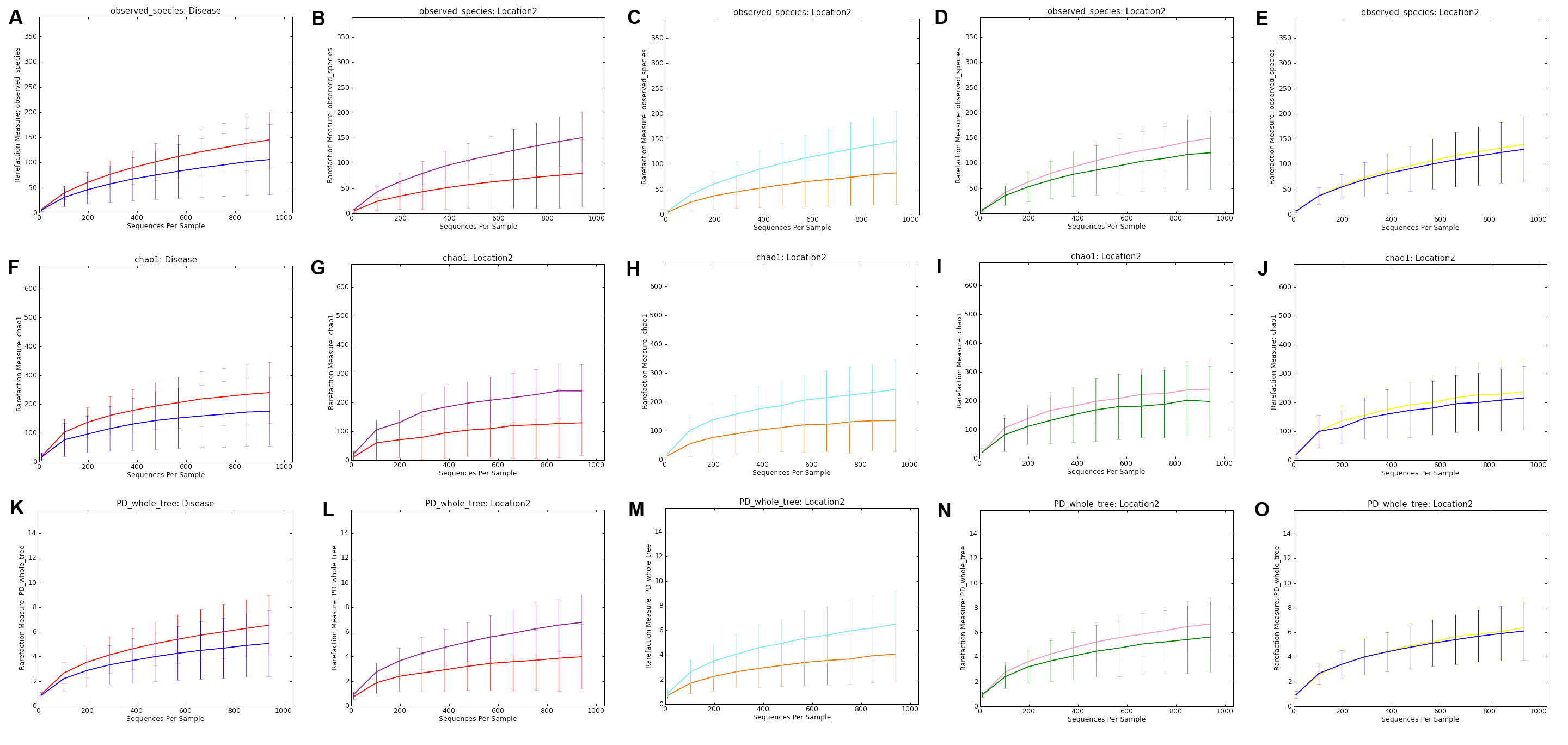

Supplement: Figure S8 — Alpha diversity assessed by diversity indices in samples without any antibiotic exposures. HIV cases versus controls are shown by sample site. HIV cases have decreased diversity compared to controls. Diversity indices shown are OTU richness (panels (a–e)), Chao1 index (panels (f–j)), and Phylogenetic Diversity (PD) Whole Tree metric (panels (k–o)). All samples are shown in panels (a),(f),(k);and HIV samples are in blue; Control samples are in red in these panels. Samples from ileum are shown in panels (b),(g),(l); and HIV ileal samples are in red; Control ileal samples are in purple in these panels. Samples from right colon are shown in panels (c),(h),(m); and HIV right colon samples are in orange; Control right colon samples are in light blue in these panels. Samples from left colon are shown in panels (d),(i),(n); and HIV left colon samples are in green; Control left colon samples are in pink in these panels. Fecal samples are shown in panels (e),(j),(o); and HIV fecal samples are in blue; Control fecal samples are in yellow in these panels. (TIF) [file ppat.1003829.s008.tif]

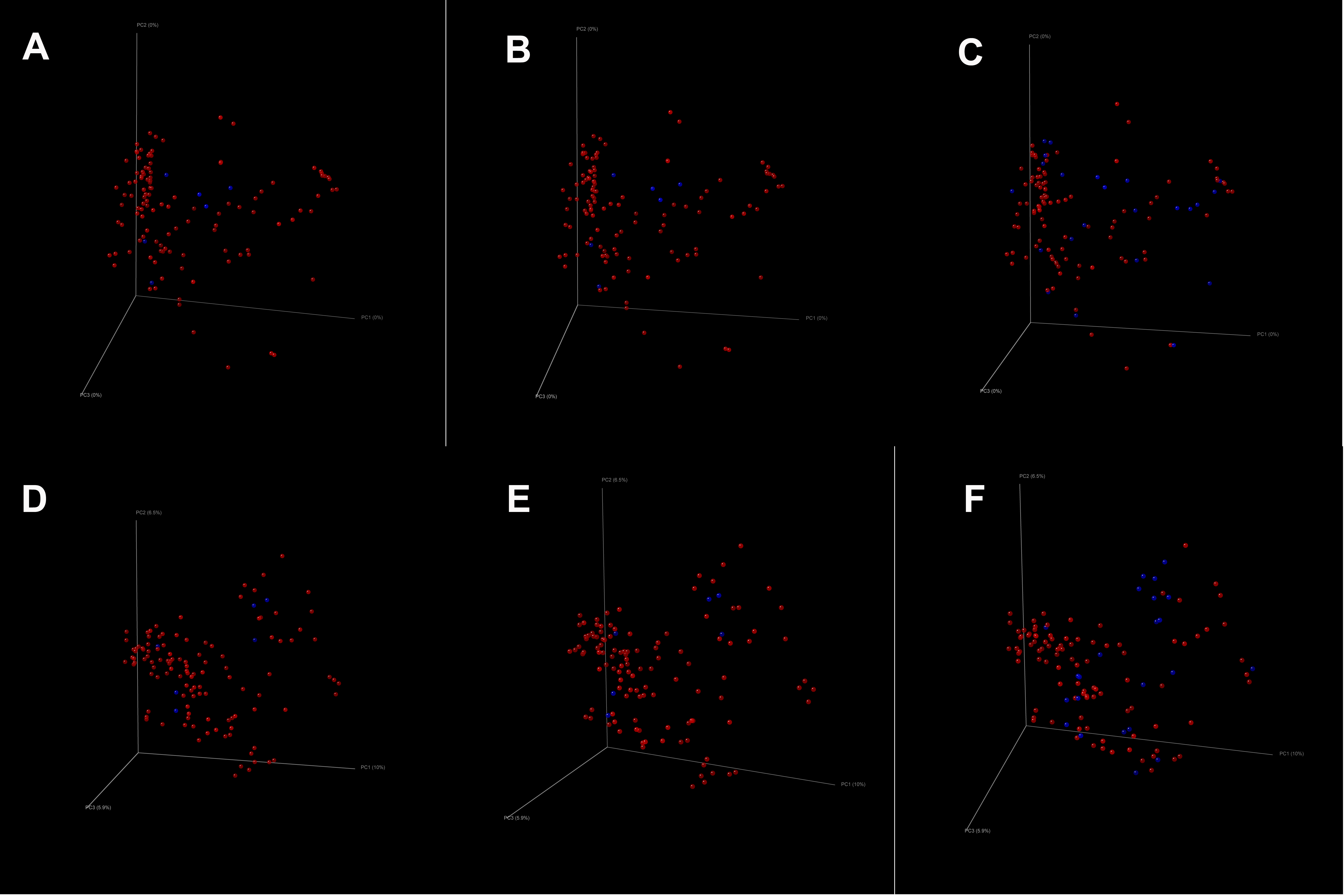

Supplement: Figure S9 — Beta diversity assessed by NMDS and PCO in samples with antibiotic exposures versus those with no antibiotic exposures. Samples collected from subjects with antibiotic exposures are shown in blue and samples collected from subjects without antibiotic exposures are shown in red. For NMDS the Bray-Curtis similarity at the OTU level was used. For PCO, Unifrac distances were used. Panels (a–c) show the results for NMDS, and panels (d–f) show the results for Unifrac. Panels (a) and (d) show samples collected from subjects within 1 month of antibiotic exposure in blue; Panels (b) and (e) show samples collected from subjects within 3 months of antibiotic exposure in blue; Panels (c) and (f) show samples collected from subjects within 1 year of antibiotic exposure in blue. No differences are noted in beta diversity. (TIF) [file ppat.1003829.s009.tif]

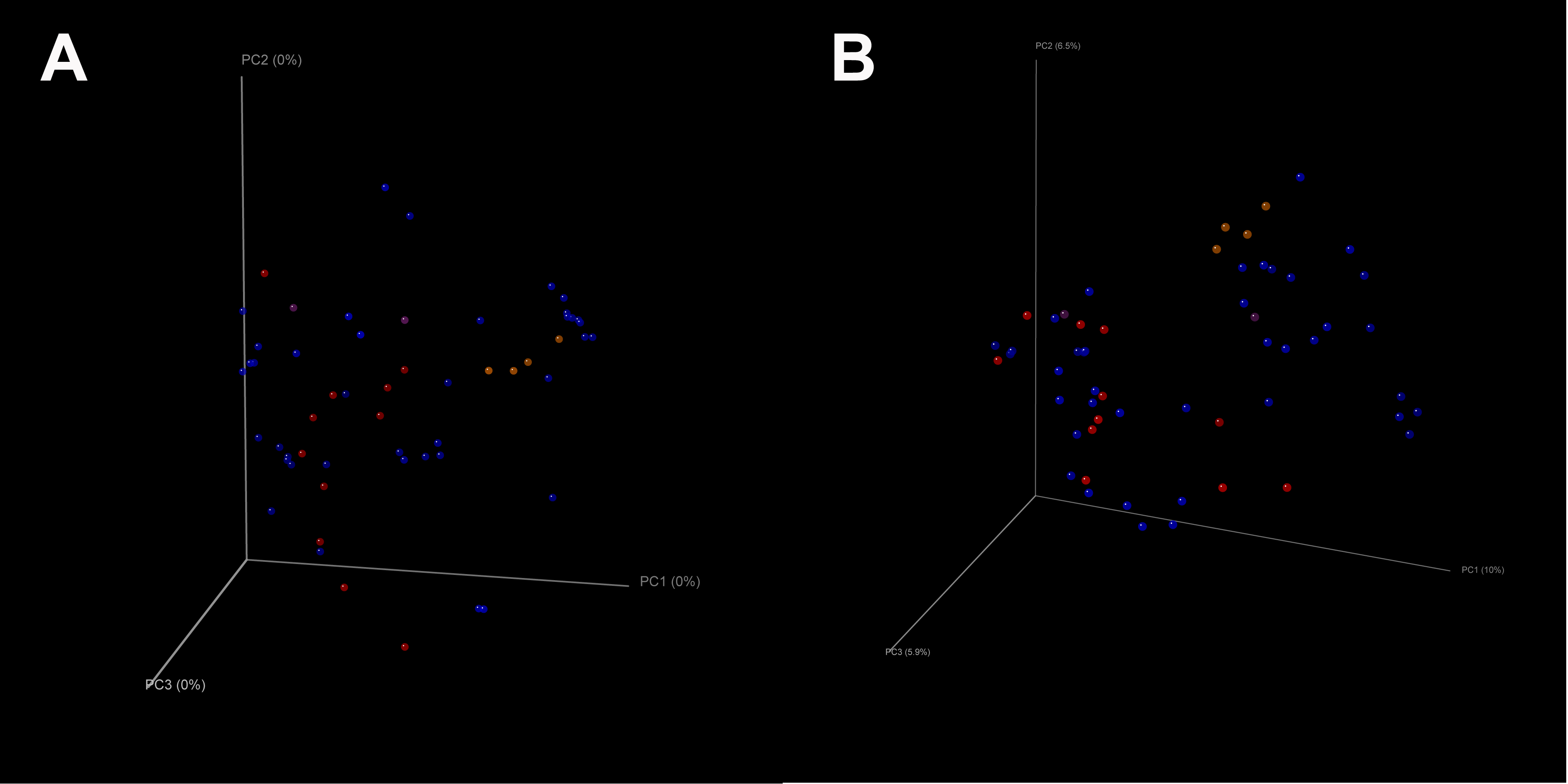

Supplement: Figure S10 — Beta diversity assessed by NMDS and PCO in samples from illicit drug users versus non-users. For NMDS (panel (a)), the Bray-Curtis similarity at the OTU level was used. For PCO (panel (b)), Unifrac distances were used. Samples from non-users are in blue; samples from current users are in red (n = 11); samples from former users are in orange (n = 4); samples for which no data is available are in purple (n = 2). No differences are noted in beta diversity. (TIF) [file ppat.1003829.s010.tif]

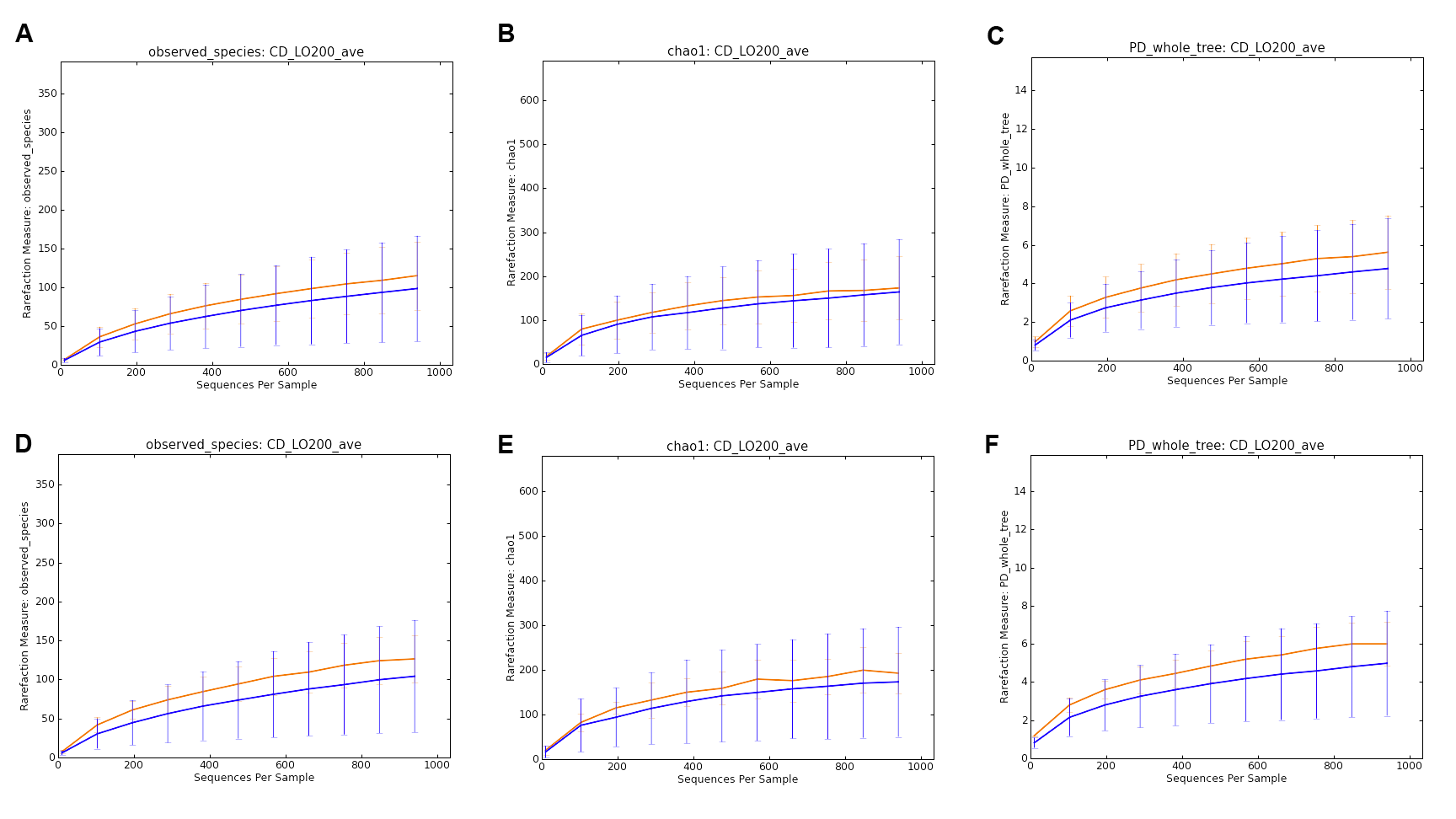

Supplement: Figure S11 — Alpha diversity assessed by diversity indices in HIV samples with CD4+ T cell counts less than 200 versus those with CD4+ T cell counts more than 200. Samples from cases with counts <200 are in orange; samples from cases with counts >200 are in blue. Panels (a–c) show results of diversity indices in all samples and panels (d–f) show results of diversity indices in samples collected from subjects without any antibiotic exposures within 1 year of mucosal sampling. Diversity indices shown are OTU richness (panels (a–d)), Chao1 index (panels (b–e)), and Phylogenetic Diversity (PD) Whole Tree metric (panels (c–f)). No differences are noted. (TIF) [file ppat.1003829.s011.tif]

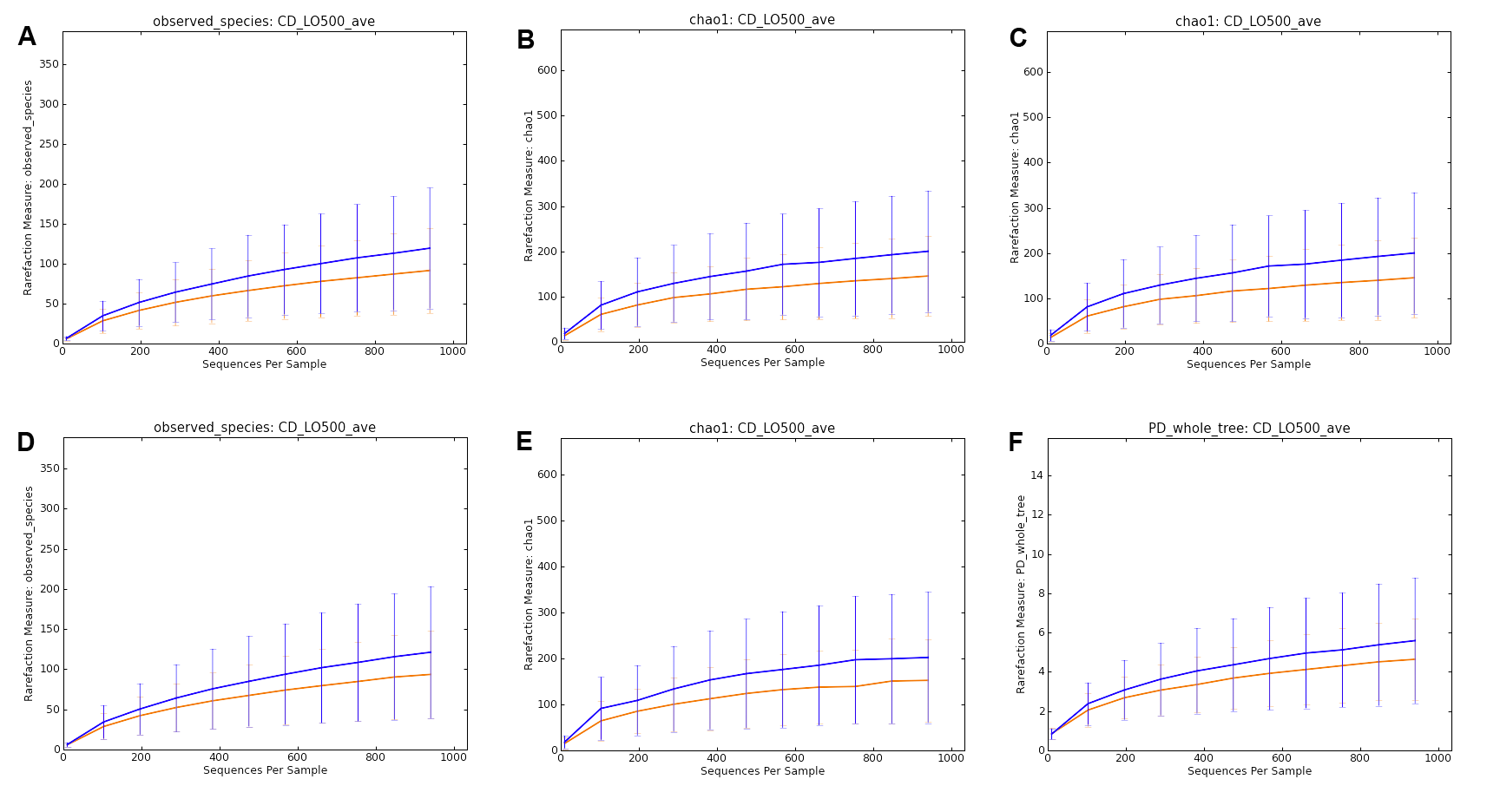

Supplement: Figure S12 — Alpha diversity assessed by diversity indices in HIV samples with CD4+ T cell counts less than 500 versus those with CD4+ T cell counts more than 500. Samples from cases with counts <500 are in orange; samples from cases with counts >500 are in blue. Panels (a–c) show results of diversity indices in all samples and panels (d–f) show results of diversity indices in samples collected from subjects without any antibiotic exposures within 1 year of mucosal sampling. Diversity indices shown are OTU richness (panels (a–d)), Chao1 index (panels (b–e)), and Phylogenetic Diversity (PD) Whole Tree metric (panels (c–f)). No differences are noted. (TIF) [file ppat.1003829.s012.tif]

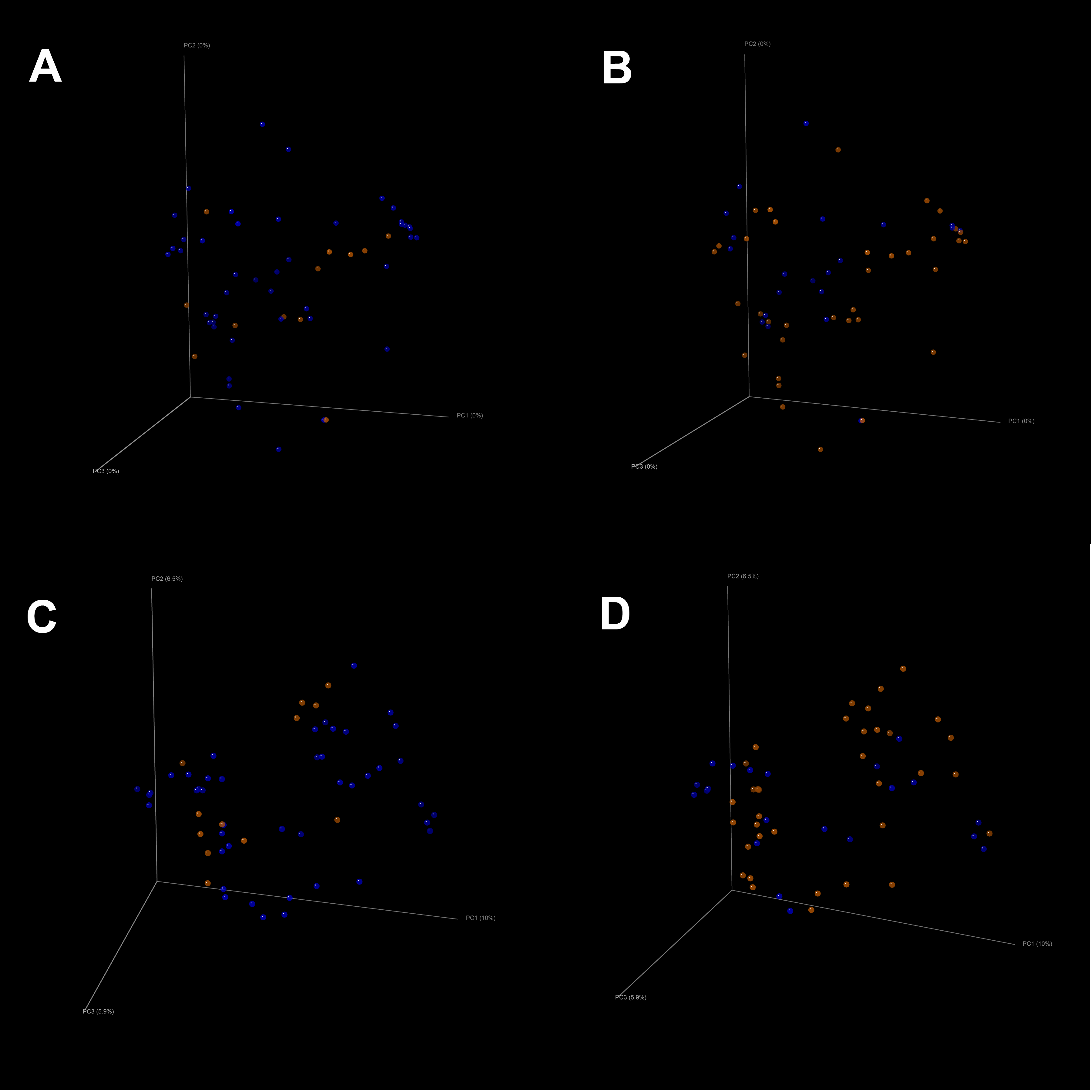

Supplement: Figure S13 — Beta diversity assessed by NMDS and PCO in samples with CD varying CD4+ T cell count thresholds. In panels (a) and (c), samples from cases with counts <200 are in orange; samples from cases with counts >200 are in blue. In panels (b) and (d), samples from cases with counts <500 are in orange; samples from cases with counts >500 are in blue. For NMDS shown in panels (a) and (b), the Bray-Curtis similarity at the OTU level was used. For PCO shown in panels (c) and (d), Unifrac distances were used. No differences are noted in beta diversity. (TIF) [file ppat.1003829.s013.tif]

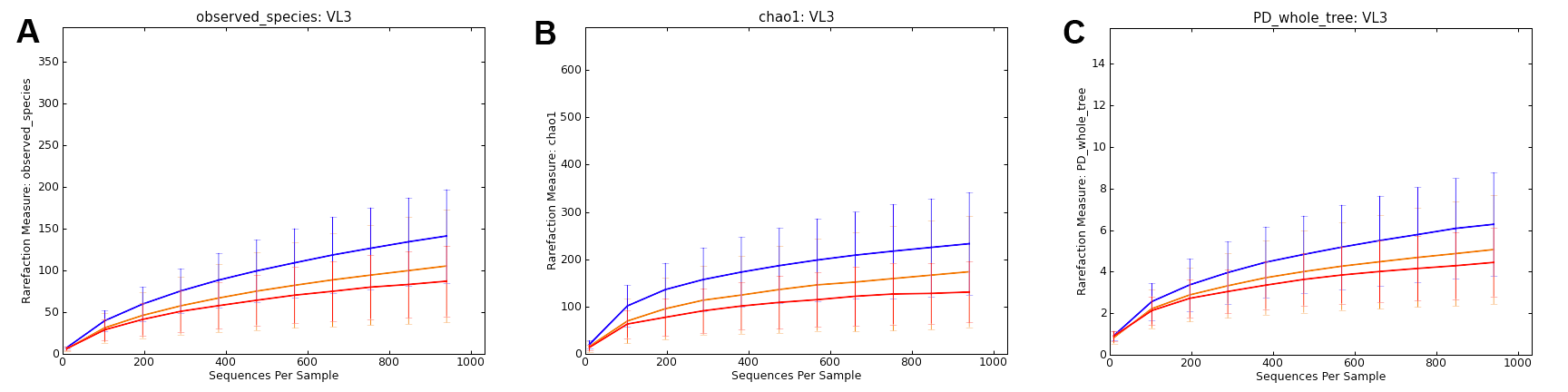

Supplement: Figure S14 — Alpha diversity assessed by diversity indices in HIV samples with detectable viremia versus those with viral suppression. Samples from controls are in blue; samples from HIV infected subjects with viremia are in red; and samples from HIV infected subjects who have viral suppression are in orange. Diversity indices shown are OTU richness (panel (a)), Chao1 index (panel (b)), and Phylogenetic Diversity (PD) Whole Tree metric (panel (c)). No differences are noted. (TIF) [file ppat.1003829.s014.tif]

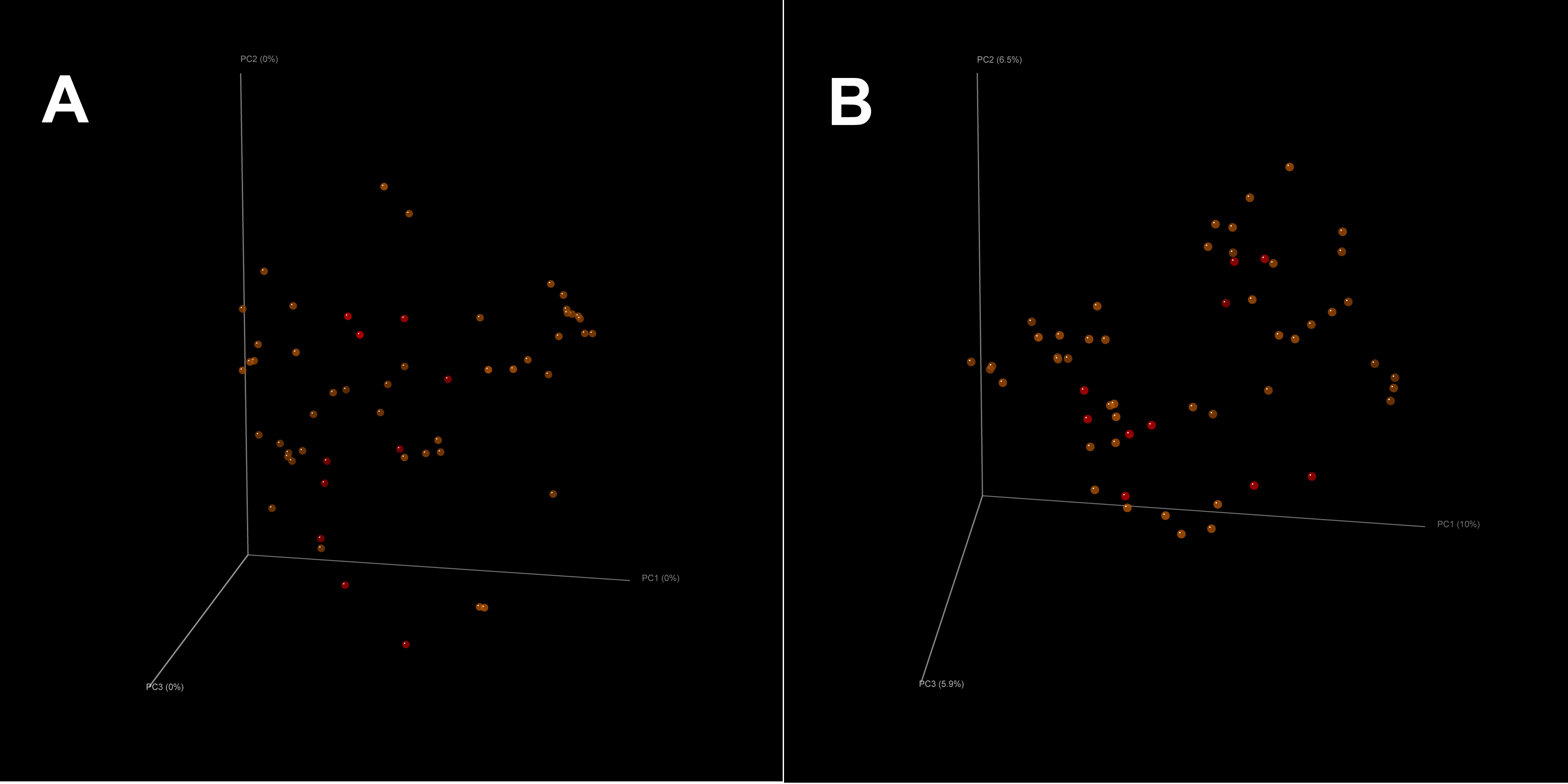

Supplement: Figure S15 — Beta diversity assessed by NMDS and PCO in samples from HIV infected subjects with and without viremia. Samples from HIV infected subjects with viremia are in red; and samples from HIV infected subjects who have viral suppression are in orange. For NMDS shown in panel (a), the Bray-Curtis similarity at the OTU level was used. For PCO shown in panels (b), Unifrac distances were used. No differences are noted in beta diversity. (TIF) [file ppat.1003829.s015.tif]

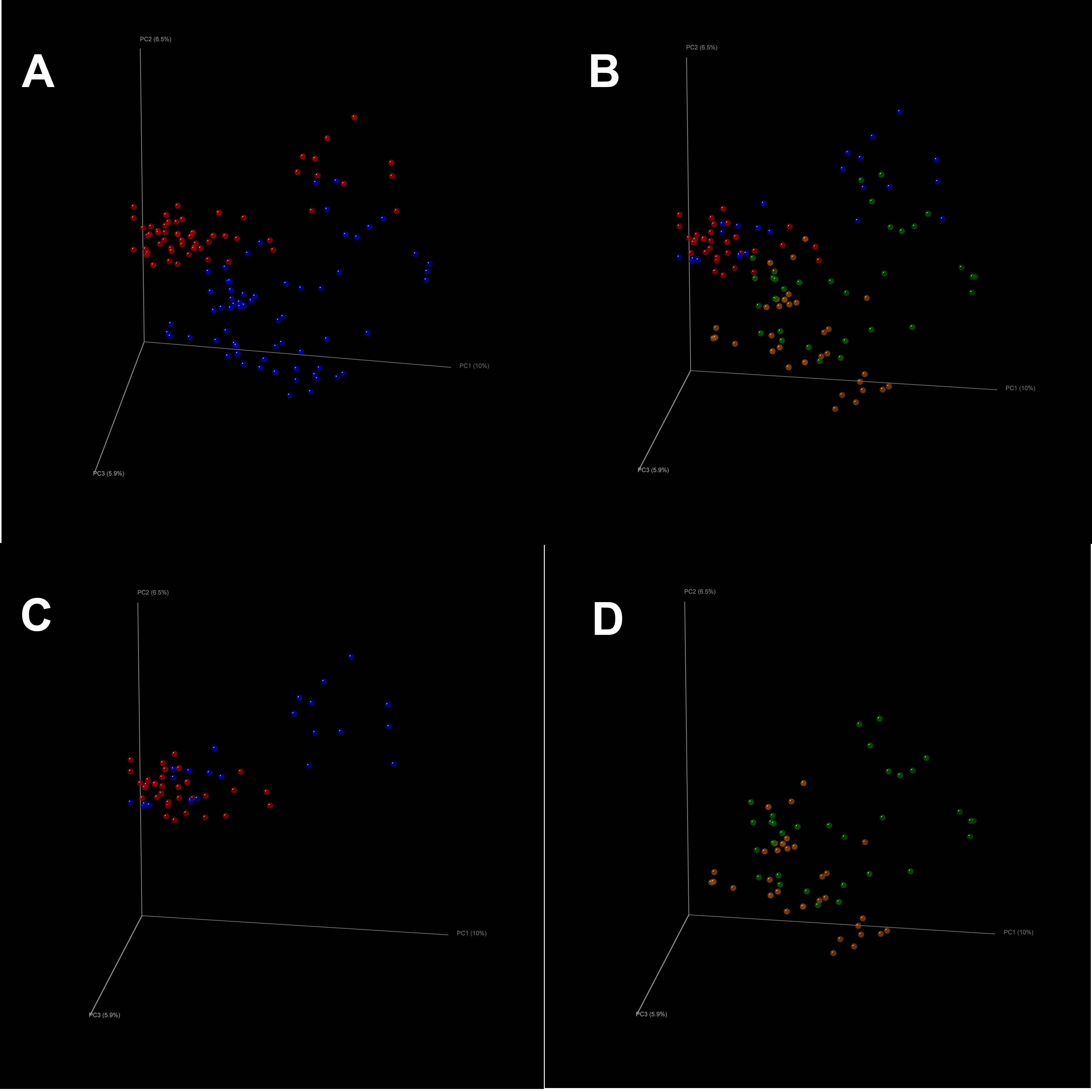

Supplement: Figure S16 — Beta diversity assessed by PCO using Unifrac distances for samples in different runs. In panel (a), samples in Run 1 are in red; and samples in Run 2 are in blue. In panel (b–d), control samples in Run 1 are shown in red; HIV samples in Run 1 are shown in blue, control samples in Run 2 are shown in orange, and HIV samples in Run 2 are shown in green. Panel (b) shows results for both runs. Panel (c) shows results for Run 1 by disease presence. Panel (d) shows results for Run 2 by disease presence. Separation of HIV and HC samples are seen in both runs when examined individually in panels (c) and (d). Run effects are in a vertical direction along axis 2 and disease effects are in a horizontal direction along axis 1, suggesting little to no effect of batch related variability in the results. (TIF) [file ppat.1003829.s016.tif]

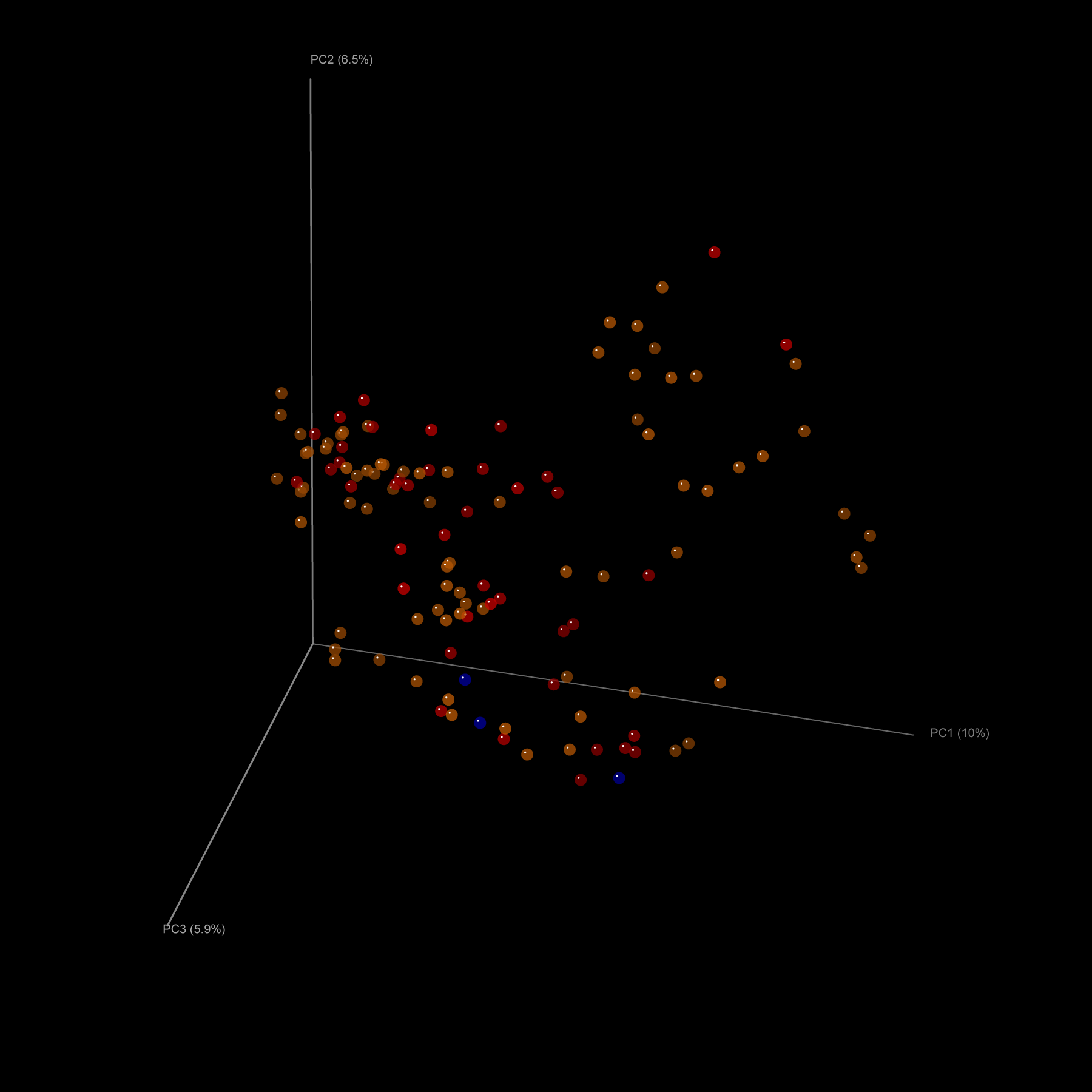

Supplement: Figure S17 — Beta diversity assessed by PCO using Unifrac distances in samples with various bowel preps. Samples from subjects taking PEG+vitC (i.e. Moviprep) are in red (n = 41); samples from subjects taking PEG are in orange (n = 77); and samples from subjects taking sodium phosphate tablets are in blue (n = 3). No differences are noted in beta diversity. (TIF) [file ppat.1003829.s017.tif]
