# Supplementary material for: A Compositional Look at the Human Gastrointestinal Microbiome and Immune Activation Parameters in HIV Infected Subjects
Source: PLoS Pathog. 2014 Feb 20;10(2):e1003829. doi: 10.1371/journal.ppat.1003829 (PMC3930561; doi:10.1371/journal.ppat.1003829)
Supplement: Table S1 — Demographics of HIV and control subjects. (DOCX) [file ppat.1003829.s018.docx]

**Table S1.** Demographics of HIV and control subjects

|  | **HIV (n=21 )** | **Controls (n= 22)** | **p-value** |
| --- | --- | --- | --- |
| **Age (Mean +/- StdDev in years)** | 52.19 **+/-** 5.2 | 54.82**+/-** 6.7 | 0.725 |
| **Gender (M/F*)** | 16/5 | 17/5 | 0.935 |
| **Race** |  |  | 0.28 |
| African American(n,%) | 10(47.6) | 13(59.1) |  |
| Asian(n,%) | 1(4.7) | 2(9.1) |  |
| Native American(n,%) | 0(0) | 1(4.7) |  |
| White(n,%) | 10(19) | 6(27.3) |  |
| **Ethnicity** |  |  | 0.349 |
| Hispanic n,% | 4(9.5) | 7(31.8) |  |
| Not hispanic n,% | 17(81) | 15(68.2) |  |

*M=male, F=female
