# Supplementary material for: A Compositional Look at the Human Gastrointestinal Microbiome and Immune Activation Parameters in HIV Infected Subjects
Source: PLoS Pathog. 2014 Feb 20;10(2):e1003829. doi: 10.1371/journal.ppat.1003829 (PMC3930561; doi:10.1371/journal.ppat.1003829)
Supplement: Table S2 — Clinical characteristics of HIV Subjects. (DOCX) [file ppat.1003829.s019.docx]

**Table S2.** Clinical Characteristics of HIV Subjects

| **Subject Number** | **CD4 count** | **Viral Load** | **Current antibiotic use** | **Current ART** |
| --- | --- | --- | --- | --- |
| 1 | 535 | <75 | No | Atazanavir/r, Emtricitabine/tenofovir disoproxil fumarate |
| 2 | 334 | 1094 | No | Atazanavir/r, Emtricitabine/tenofovir disoproxil fumarate |
| 3 | 867 | 407 | No | None |
| 4 | 124 | <75 | No | Atazanavir/r, Emtricitabine/tenofovir disoproxil fumarate |
| 5 | 233 | <75 | No | Efavirenz/emtricitabine/tenofovir disoproxil fumarate |
| 6 | 790 | <75 | No | Fosamprenavir/r, Emtricitabine/tenofovir disoproxil fumarate |
| 7 | 267 | <75 | No | Efavirenz/emtricitabine/tenofovir disoproxil fumarate |
| 8 | 206 | <75 | No | Atazanavir/r, Emtricitabine/tenofovir disoproxil fumarate |
| 9 | 713 | <75 | No | None |
| 10 | 350 | <40 | No | Atazanavir/r, Emtricitabine/tenofovir disoproxil fumarate |
| 11 | 321 | <75 | No | Efavirenz/emtricitabine/tenofovir disoproxil fumarate |
| 12 | 948 | <40 | No | Efavirenz/emtricitabine/tenofovir disoproxil fumarate |
| 13 | 631 | <40 | No | Efavirenz/emtricitabine/tenofovir disoproxil fumarate |
| 14 | 534 | <40 | No | Efavirenz/emtricitabine/tenofovir disoproxil fumarate |
| 15 | 565 | 179 | No | Efavirenz/emtricitabine/tenofovir disoproxil fumarate |
| 16 | 122 | <40 | Trimethoprim/  Sulfamethoxazole | Raltegravir ,emtricitabine/tenofovir disoproxil fumarate |
| 17 | 314 | <40 | No | Efavirenz/emtricitabine/tenofovir disoproxil fumarate, Atazanavir/r |
| 18 | 106 | 4604 | No | Rilpivirine ,Emtricitabine/tenofovir disoproxil fumarate |
| 19 | 206 | <40 | No | Fosamprenavir/r, Emtricitabine/tenofovir disoproxil fumarate |
| 20 | 597 | <40 | No | Darunavir/r ,Raltegravir ,Emtricitabine/tenofovir disoproxil fumarate |
| 21 | 167 | <40 | No | Efavirenz/emtricitabine/tenofovir disoproxil fumarate |
