# Supplementary material for: A Compositional Look at the Human Gastrointestinal Microbiome and Immune Activation Parameters in HIV Infected Subjects
Source: PLoS Pathog. 2014 Feb 20;10(2):e1003829. doi: 10.1371/journal.ppat.1003829 (PMC3930561; doi:10.1371/journal.ppat.1003829)
Supplement: Table S3 — Network statistics for the overall bipartite sample and OTU network. (DOCX) [file ppat.1003829.s020.docx]

**Table S3. Network statistics for the overall bipartite sample and OTU network**

| **Network parameter** | **Overall**  **network** | **HIV Nodes Subnetwork** | **Control Nodes**  **Subnetwork** | **All Sample Nodes Subnetwork** |
| --- | --- | --- | --- | --- |
| Network diameter | 6 |  |  |  |
| Network radius | 3 |  |  |  |
| Clustering coefficient | 0 | 0 | 0 | 0 |
| Network centralization | 0.094 | 2.988 | 2.332 | 1.382 |
| Characteristic path length | 3.643 |  |  |  |
| Shortest paths | 8,535,162(100%) |  |  |  |
| Average number of neighbors | 10.181 | 101.554 | 141.338 | 122.926 |
| Number of nodes | 2922 | 56 | 65 | 121 |
| Network density | 0.003 | 1.846 | 2.208 | 1.024 |
| Network heterogeneity | 2.792 | 0.634 | 0.402 | 0.517 |
| Isolated nodes | 0 | 0 | 0 | 0 |
| Number of self-loops | 0 | 0 | 0 | 0 |
| Multi-edge node pairs | 0 | 0 | 0 | 0 |
| Connected components | 1 | 1 | 1 | 1 |
