# Supplementary material for: A Compositional Look at the Human Gastrointestinal Microbiome and Immune Activation Parameters in HIV Infected Subjects
Source: PLoS Pathog. 2014 Feb 20;10(2):e1003829. doi: 10.1371/journal.ppat.1003829 (PMC3930561; doi:10.1371/journal.ppat.1003829)
Supplement: Table S4 — Phyla level bacterial microbiome composition in control and HIV samples. (DOCX) [file ppat.1003829.s021.docx]

**Table S4.** Phyla level bacterial microbiome composition in control and HIV samples

| **Taxon** | **Control** |  | **HIV** |  |
| --- | --- | --- | --- | --- |
|  | **Mean** | **SD** | **Mean** | **SD** |
| k__Bacteria;p__Acidobacteria | 0.0000 | 0.0001 | 0.0001 | 0.0002 |
| k__Bacteria;p__Actinobacteria | 0.0008 | 0.0023 | 0.0010 | 0.0023 |
| k__Bacteria;p__Bacteroidetes | 0.2860 | 0.1793 | 0.2120 | 0.1922 |
| k__Bacteria;p__Chloroflexi | 0.0001 | 0.0008 | 0.0000 | 0.0000 |
| k__Bacteria;p__Cyanobacteria | 0.0011 | 0.0026 | 0.0003 | 0.0008 |
| k__Bacteria;p__Elusimicrobia | 0.0000 | 0.0000 | 0.0001 | 0.0002 |
| k__Bacteria;p__Firmicutes | 0.6349 | 0.1730 | 0.3740 | 0.2729 |
| k__Bacteria;p__Fusobacteria | 0.0154 | 0.0664 | 0.0096 | 0.0285 |
| k__Bacteria;p__PAUC34f | 0.0000 | 0.0003 | 0.0000 | 0.0000 |
| k__Bacteria;p__Planctomycetes | 0.0000 | 0.0001 | 0.0000 | 0.0000 |
| k__Bacteria;p__Proteobacteria | 0.0529 | 0.0998 | 0.3288 | 0.3671 |
| k__Bacteria;p__SBR1093 | 0.0000 | 0.0000 | 0.0000 | 0.0001 |
| k__Bacteria;p__Spirochaetes | 0.0000 | 0.0000 | 0.0738 | 0.2404 |
| k__Bacteria;p__Synergistetes | 0.0003 | 0.0022 | 0.0001 | 0.0003 |
| k__Bacteria;p__TM7 | 0.0000 | 0.0000 | 0.0000 | 0.0003 |
| k__Bacteria;p__Tenericutes | 0.0001 | 0.0005 | 0.0001 | 0.0005 |
| k__Bacteria;p__Verrucomicrobia | 0.0083 | 0.0261 | 0.0003 | 0.0013 |
