# Supplementary material for: A Compositional Look at the Human Gastrointestinal Microbiome and Immune Activation Parameters in HIV Infected Subjects
Source: PLoS Pathog. 2014 Feb 20;10(2):e1003829. doi: 10.1371/journal.ppat.1003829 (PMC3930561; doi:10.1371/journal.ppat.1003829)
Supplement: Table S5 — Class level bacterial microbiome composition in control and HIV samples. (DOCX) [file ppat.1003829.s022.docx]

**Table S5.** Class level bacterial microbiome composition in control and HIV samples

| **Taxon** | **Control** |  | **HIV** |  |
| --- | --- | --- | --- | --- |
|  | **Mean** | **SD** | **Mean** | **SD** |
| k__Bacteria;p__Acidobacteria;c__Acidobacteria-2 | 0.0000 | 0.0000 | 0.0001 | 0.0002 |
| k__Bacteria;p__Acidobacteria;c__Sva0725 | 0.0000 | 0.0001 | 0.0000 | 0.0000 |
| k__Bacteria;p__Actinobacteria;c__Acidimicrobiia | 0.0000 | 0.0000 | 0.0000 | 0.0001 |
| k__Bacteria;p__Actinobacteria;c__Actinobacteria | 0.0008 | 0.0023 | 0.0010 | 0.0023 |
| k__Bacteria;p__Bacteroidetes;Other | 0.0001 | 0.0003 | 0.0000 | 0.0000 |
| k__Bacteria;p__Bacteroidetes;c__Bacteroidia | 0.2843 | 0.1807 | 0.2120 | 0.1922 |
| k__Bacteria;p__Bacteroidetes;c__Flavobacteriia | 0.0011 | 0.0078 | 0.0000 | 0.0000 |
| k__Bacteria;p__Bacteroidetes;c__Sphingobacteriia | 0.0005 | 0.0032 | 0.0000 | 0.0000 |
| k__Bacteria;p__Chloroflexi;c__Anaerolineae | 0.0001 | 0.0008 | 0.0000 | 0.0000 |
| k__Bacteria;p__Cyanobacteria;c__4C0d-2 | 0.0002 | 0.0008 | 0.0000 | 0.0001 |
| k__Bacteria;p__Cyanobacteria;c__Chloroplast | 0.0002 | 0.0006 | 0.0001 | 0.0004 |
| k__Bacteria;p__Cyanobacteria;c__S15B-MN24 | 0.0001 | 0.0005 | 0.0002 | 0.0006 |
| k__Bacteria;p__Cyanobacteria;c__Synechococcophycideae | 0.0007 | 0.0021 | 0.0000 | 0.0001 |
| k__Bacteria;p__Elusimicrobia;c__Elusimicrobia | 0.0000 | 0.0000 | 0.0001 | 0.0002 |
| k__Bacteria;p__Firmicutes;Other | 0.0001 | 0.0005 | 0.0000 | 0.0001 |
| k__Bacteria;p__Firmicutes;c__Bacilli | 0.0365 | 0.1169 | 0.0297 | 0.1197 |
| k__Bacteria;p__Firmicutes;c__Clostridia | 0.5450 | 0.1804 | 0.3044 | 0.2532 |
| k__Bacteria;p__Firmicutes;c__Erysipelotrichi | 0.0532 | 0.1153 | 0.0399 | 0.0679 |
| k__Bacteria;p__Fusobacteria;c__Fusobacteria | 0.0154 | 0.0664 | 0.0096 | 0.0285 |
| k__Bacteria;p__PAUC34f;c__ | 0.0000 | 0.0003 | 0.0000 | 0.0000 |
| k__Bacteria;p__Planctomycetes;c__Planctomycetia | 0.0000 | 0.0001 | 0.0000 | 0.0000 |
| k__Bacteria;p__Proteobacteria;Other | 0.0002 | 0.0009 | 0.0000 | 0.0000 |
| k__Bacteria;p__Proteobacteria;c__Alphaproteobacteria | 0.0007 | 0.0027 | 0.0002 | 0.0006 |
| k__Bacteria;p__Proteobacteria;c__Betaproteobacteria | 0.0163 | 0.0425 | 0.0399 | 0.1307 |
| k__Bacteria;p__Proteobacteria;c__Deltaproteobacteria | 0.0004 | 0.0012 | 0.0002 | 0.0007 |
| k__Bacteria;p__Proteobacteria;c__Epsilonproteobacteria | 0.0012 | 0.0065 | 0.0260 | 0.1136 |
| k__Bacteria;p__Proteobacteria;c__Gammaproteobacteria | 0.0341 | 0.0882 | 0.2626 | 0.3578 |
| k__Bacteria;p__SBR1093;c__EC214 | 0.0000 | 0.0000 | 0.0000 | 0.0001 |
| k__Bacteria;p__Spirochaetes;c__[Brachyspirae] | 0.0000 | 0.0000 | 0.0738 | 0.2404 |
| k__Bacteria;p__Synergistetes;c__Synergistia | 0.0003 | 0.0022 | 0.0001 | 0.0003 |
| k__Bacteria;p__TM7;c__TM7-3 | 0.0000 | 0.0000 | 0.0000 | 0.0003 |
| k__Bacteria;p__Tenericutes;c__Mollicutes | 0.0001 | 0.0005 | 0.0001 | 0.0005 |
| k__Bacteria;p__Verrucomicrobia;c__Opitutae | 0.0000 | 0.0000 | 0.0000 | 0.0003 |
| k__Bacteria;p__Verrucomicrobia;c__Verrucomicrobiae | 0.0083 | 0.0261 | 0.0002 | 0.0011 |
