# Supplementary material for: A Compositional Look at the Human Gastrointestinal Microbiome and Immune Activation Parameters in HIV Infected Subjects
Source: PLoS Pathog. 2014 Feb 20;10(2):e1003829. doi: 10.1371/journal.ppat.1003829 (PMC3930561; doi:10.1371/journal.ppat.1003829)
Supplement: Table S6 — Order level bacterial microbiome composition in control and HIV samples. (DOCX) [file ppat.1003829.s023.docx]

**Table S6.** Order level bacterial microbiome composition in control and HIV samples

| **Taxon** | **Control** |  | **HIV** |  |
| --- | --- | --- | --- | --- |
|  | **Mean** | **SD** | **Mean** | **SD** |
| k__Bacteria;p__Acidobacteria;c__Acidobacteria-2;o__ | 0.0000 | 0.0000 | 0.0001 | 0.0002 |
| k__Bacteria;p__Acidobacteria;c__Sva0725;o__Sva0725 | 0.0000 | 0.0001 | 0.0000 | 0.0000 |
| k__Bacteria;p__Actinobacteria;c__Acidimicrobiia;o__Acidimicrobiales | 0.0000 | 0.0000 | 0.0000 | 0.0001 |
| k__Bacteria;p__Actinobacteria;c__Actinobacteria;o__Actinomycetales | 0.0008 | 0.0023 | 0.0010 | 0.0023 |
| k__Bacteria;p__Bacteroidetes;Other;Other | 0.0001 | 0.0003 | 0.0000 | 0.0000 |
| k__Bacteria;p__Bacteroidetes;c__Bacteroidia;o__Bacteroidales | 0.2843 | 0.1807 | 0.2120 | 0.1922 |
| k__Bacteria;p__Bacteroidetes;c__Flavobacteriia;o__ | 0.0000 | 0.0002 | 0.0000 | 0.0000 |
| k__Bacteria;p__Bacteroidetes;c__Flavobacteriia;o__Flavobacteriales | 0.0011 | 0.0078 | 0.0000 | 0.0000 |
| k__Bacteria;p__Bacteroidetes;c__Sphingobacteriia;o__Sphingobacteriales | 0.0005 | 0.0032 | 0.0000 | 0.0000 |
| k__Bacteria;p__Chloroflexi;c__Anaerolineae;o__SBR1031 | 0.0001 | 0.0008 | 0.0000 | 0.0000 |
| k__Bacteria;p__Cyanobacteria;c__4C0d-2;o__MLE1-12 | 0.0000 | 0.0001 | 0.0000 | 0.0000 |
| k__Bacteria;p__Cyanobacteria;c__4C0d-2;o__YS2 | 0.0002 | 0.0008 | 0.0000 | 0.0001 |
| k__Bacteria;p__Cyanobacteria;c__Chloroplast;Other | 0.0001 | 0.0004 | 0.0000 | 0.0000 |
| k__Bacteria;p__Cyanobacteria;c__Chloroplast;o__Chlorophyta | 0.0000 | 0.0004 | 0.0000 | 0.0000 |
| k__Bacteria;p__Cyanobacteria;c__Chloroplast;o__Streptophyta | 0.0000 | 0.0003 | 0.0001 | 0.0004 |
| k__Bacteria;p__Cyanobacteria;c__S15B-MN24;o__ | 0.0001 | 0.0005 | 0.0002 | 0.0006 |
| k__Bacteria;p__Cyanobacteria;c__Synechococcophycideae;o__Synechococcales | 0.0007 | 0.0021 | 0.0000 | 0.0001 |
| k__Bacteria;p__Elusimicrobia;c__Elusimicrobia;o__Elusimicrobiales | 0.0000 | 0.0000 | 0.0001 | 0.0002 |
| k__Bacteria;p__Firmicutes;Other;Other | 0.0001 | 0.0005 | 0.0000 | 0.0001 |
| k__Bacteria;p__Firmicutes;c__Bacilli;Other | 0.0010 | 0.0050 | 0.0008 | 0.0025 |
| k__Bacteria;p__Firmicutes;c__Bacilli;o__ | 0.0000 | 0.0001 | 0.0000 | 0.0000 |
| k__Bacteria;p__Firmicutes;c__Bacilli;o__Bacillales | 0.0005 | 0.0018 | 0.0000 | 0.0001 |
| k__Bacteria;p__Firmicutes;c__Bacilli;o__Gemellales | 0.0007 | 0.0024 | 0.0002 | 0.0008 |
| k__Bacteria;p__Firmicutes;c__Bacilli;o__Lactobacillales | 0.0343 | 0.1140 | 0.0286 | 0.1179 |
| k__Bacteria;p__Firmicutes;c__Bacilli;o__Turicibacterales | 0.0001 | 0.0003 | 0.0000 | 0.0003 |
| k__Bacteria;p__Firmicutes;c__Clostridia;Other | 0.0019 | 0.0024 | 0.0013 | 0.0020 |
| k__Bacteria;p__Firmicutes;c__Clostridia;o__ | 0.0013 | 0.0025 | 0.0025 | 0.0051 |
| k__Bacteria;p__Firmicutes;c__Clostridia;o__Clostridiales | 0.5401 | 0.1797 | 0.2979 | 0.2503 |
| k__Bacteria;p__Firmicutes;c__Clostridia;o__Coriobacteriales | 0.0017 | 0.0030 | 0.0027 | 0.0043 |
| k__Bacteria;p__Firmicutes;c__Erysipelotrichi;o__Erysipelotrichales | 0.0532 | 0.1153 | 0.0399 | 0.0679 |
| k__Bacteria;p__Fusobacteria;c__Fusobacteria;o__Fusobacteriales | 0.0154 | 0.0664 | 0.0096 | 0.0285 |
| k__Bacteria;p__PAUC34f;c__;o__ | 0.0000 | 0.0003 | 0.0000 | 0.0000 |
| k__Bacteria;p__Planctomycetes;c__Planctomycetia;o__Pirellulales | 0.0000 | 0.0001 | 0.0000 | 0.0000 |
| k__Bacteria;p__Proteobacteria;Other;Other | 0.0002 | 0.0009 | 0.0000 | 0.0000 |
| k__Bacteria;p__Proteobacteria;c__Alphaproteobacteria;o__Caulobacterales | 0.0000 | 0.0003 | 0.0000 | 0.0001 |
| k__Bacteria;p__Proteobacteria;c__Alphaproteobacteria;o__RF32 | 0.0000 | 0.0003 | 0.0001 | 0.0004 |
| k__Bacteria;p__Proteobacteria;c__Alphaproteobacteria;o__Rhizobiales | 0.0001 | 0.0007 | 0.0001 | 0.0004 |
| k__Bacteria;p__Proteobacteria;c__Alphaproteobacteria;o__Rhodobacterales | 0.0002 | 0.0016 | 0.0000 | 0.0000 |
| k__Bacteria;p__Proteobacteria;c__Alphaproteobacteria;o__Rhodospirillales | 0.0000 | 0.0001 | 0.0000 | 0.0000 |
| k__Bacteria;p__Proteobacteria;c__Alphaproteobacteria;o__Rickettsiales | 0.0002 | 0.0009 | 0.0000 | 0.0000 |
| k__Bacteria;p__Proteobacteria;c__Alphaproteobacteria;o__Sphingomonadales | 0.0001 | 0.0008 | 0.0000 | 0.0000 |
| k__Bacteria;p__Proteobacteria;c__Betaproteobacteria;o__Burkholderiales | 0.0160 | 0.0421 | 0.0391 | 0.1302 |
| k__Bacteria;p__Proteobacteria;c__Betaproteobacteria;o__Neisseriales | 0.0003 | 0.0018 | 0.0007 | 0.0025 |
| k__Bacteria;p__Proteobacteria;c__Betaproteobacteria;o__Procabacteriales | 0.0001 | 0.0005 | 0.0000 | 0.0003 |
| k__Bacteria;p__Proteobacteria;c__Deltaproteobacteria;o__Bdellovibrionales | 0.0000 | 0.0003 | 0.0000 | 0.0000 |
| k__Bacteria;p__Proteobacteria;c__Deltaproteobacteria;o__Desulfovibrionales | 0.0003 | 0.0009 | 0.0002 | 0.0007 |
| k__Bacteria;p__Proteobacteria;c__Deltaproteobacteria;o__GMD14H09 | 0.0000 | 0.0003 | 0.0000 | 0.0000 |
| k__Bacteria;p__Proteobacteria;c__Deltaproteobacteria;o__Myxococcales | 0.0000 | 0.0004 | 0.0000 | 0.0000 |
| k__Bacteria;p__Proteobacteria;c__Epsilonproteobacteria;o__Campylobacterales | 0.0012 | 0.0065 | 0.0260 | 0.1136 |
| k__Bacteria;p__Proteobacteria;c__Gammaproteobacteria;Other | 0.0000 | 0.0003 | 0.0000 | 0.0002 |
| k__Bacteria;p__Proteobacteria;c__Gammaproteobacteria;o__Aeromonadales | 0.0000 | 0.0000 | 0.0001 | 0.0002 |
| k__Bacteria;p__Proteobacteria;c__Gammaproteobacteria;o__Alteromonadales | 0.0000 | 0.0002 | 0.0000 | 0.0000 |
| k__Bacteria;p__Proteobacteria;c__Gammaproteobacteria;o__Chromatiales | 0.0000 | 0.0001 | 0.0000 | 0.0000 |
| k__Bacteria;p__Proteobacteria;c__Gammaproteobacteria;o__Enterobacteriales | 0.0288 | 0.0888 | 0.2564 | 0.3566 |
| k__Bacteria;p__Proteobacteria;c__Gammaproteobacteria;o__Oceanospirillales | 0.0001 | 0.0005 | 0.0000 | 0.0000 |
| k__Bacteria;p__Proteobacteria;c__Gammaproteobacteria;o__Pasteurellales | 0.0045 | 0.0095 | 0.0056 | 0.0112 |
| k__Bacteria;p__Proteobacteria;c__Gammaproteobacteria;o__Pseudomonadales | 0.0007 | 0.0021 | 0.0005 | 0.0015 |
| k__Bacteria;p__SBR1093;c__EC214;o__ | 0.0000 | 0.0000 | 0.0000 | 0.0001 |
| k__Bacteria;p__Spirochaetes;c__[Brachyspirae];o__[Brachyspirales] | 0.0000 | 0.0000 | 0.0738 | 0.2404 |
| k__Bacteria;p__Synergistetes;c__Synergistia;o__Synergistales | 0.0003 | 0.0022 | 0.0001 | 0.0003 |
| k__Bacteria;p__TM7;c__TM7-3;o__ | 0.0000 | 0.0000 | 0.0000 | 0.0003 |
| k__Bacteria;p__Tenericutes;c__Mollicutes;o__Anaeroplasmatales | 0.0000 | 0.0000 | 0.0001 | 0.0005 |
| k__Bacteria;p__Tenericutes;c__Mollicutes;o__Mycoplasmatales | 0.0000 | 0.0002 | 0.0000 | 0.0000 |
| k__Bacteria;p__Tenericutes;c__Mollicutes;o__RF39 | 0.0001 | 0.0004 | 0.0000 | 0.0000 |
| k__Bacteria;p__Verrucomicrobia;c__Opitutae;o__[Cerasicoccales] | 0.0000 | 0.0000 | 0.0000 | 0.0003 |
| k__Bacteria;p__Verrucomicrobia;c__Verrucomicrobiae;o__Verrucomicrobiales | 0.0083 | 0.0261 | 0.0002 | 0.0011 |
