# Supplementary material for: A Compositional Look at the Human Gastrointestinal Microbiome and Immune Activation Parameters in HIV Infected Subjects
Source: PLoS Pathog. 2014 Feb 20;10(2):e1003829. doi: 10.1371/journal.ppat.1003829 (PMC3930561; doi:10.1371/journal.ppat.1003829)
Supplement: Table S7 — Family level bacterial microbiome composition in control and HIV samples. (DOCX) [file ppat.1003829.s024.docx]

**Table S7.** Family level bacterial microbiome composition in control and HIV samples

| **Taxon** | **Control** |  | **HIV** |  |
| --- | --- | --- | --- | --- |
|  | **Mean** | **SD** | **Mean** | **SD** |
| k__Bacteria;p__Acidobacteria;c__Acidobacteria-2;o__;f__ | 0.0000 | 0.0000 | 0.0001 | 0.0002 |
| k__Bacteria;p__Acidobacteria;c__Sva0725;o__Sva0725;f__ | 0.0000 | 0.0001 | 0.0000 | 0.0000 |
| k__Bacteria;p__Actinobacteria;c__Acidimicrobiia;o__Acidimicrobiales;f__koll13 | 0.0000 | 0.0000 | 0.0000 | 0.0001 |
| k__Bacteria;p__Actinobacteria;c__Actinobacteria;o__Actinomycetales;Other | 0.0000 | 0.0000 | 0.0001 | 0.0005 |
| k__Bacteria;p__Actinobacteria;c__Actinobacteria;o__Actinomycetales;f__Actinomycetaceae | 0.0006 | 0.0021 | 0.0004 | 0.0011 |
| k__Bacteria;p__Actinobacteria;c__Actinobacteria;o__Actinomycetales;f__Brevibacteriaceae | 0.0000 | 0.0003 | 0.0000 | 0.0000 |
| k__Bacteria;p__Actinobacteria;c__Actinobacteria;o__Actinomycetales;f__Corynebacteriaceae | 0.0000 | 0.0002 | 0.0001 | 0.0002 |
| k__Bacteria;p__Actinobacteria;c__Actinobacteria;o__Actinomycetales;f__Micrococcaceae | 0.0001 | 0.0004 | 0.0001 | 0.0007 |
| k__Bacteria;p__Actinobacteria;c__Actinobacteria;o__Actinomycetales;f__Nocardiaceae | 0.0000 | 0.0003 | 0.0000 | 0.0000 |
| k__Bacteria;p__Actinobacteria;c__Actinobacteria;o__Actinomycetales;f__Propionibacteriaceae | 0.0001 | 0.0004 | 0.0003 | 0.0018 |
| k__Bacteria;p__Bacteroidetes;Other;Other;Other | 0.0001 | 0.0003 | 0.0000 | 0.0000 |
| k__Bacteria;p__Bacteroidetes;c__Bacteroidia;o__Bacteroidales;Other | 0.0011 | 0.0026 | 0.0026 | 0.0080 |
| k__Bacteria;p__Bacteroidetes;c__Bacteroidia;o__Bacteroidales;f__ | 0.0004 | 0.0014 | 0.0000 | 0.0000 |
| k__Bacteria;p__Bacteroidetes;c__Bacteroidia;o__Bacteroidales;f__Bacteroidaceae | 0.2362 | 0.1941 | 0.0905 | 0.1458 |
| k__Bacteria;p__Bacteroidetes;c__Bacteroidia;o__Bacteroidales;f__Porphyromonadaceae | 0.0066 | 0.0093 | 0.0273 | 0.0870 |
| k__Bacteria;p__Bacteroidetes;c__Bacteroidia;o__Bacteroidales;f__Prevotellaceae | 0.0266 | 0.0527 | 0.0717 | 0.1211 |
| k__Bacteria;p__Bacteroidetes;c__Bacteroidia;o__Bacteroidales;f__Rikenellaceae | 0.0048 | 0.0092 | 0.0026 | 0.0063 |
| k__Bacteria;p__Bacteroidetes;c__Bacteroidia;o__Bacteroidales;f__S24-7 | 0.0003 | 0.0011 | 0.0001 | 0.0003 |
| k__Bacteria;p__Bacteroidetes;c__Bacteroidia;o__Bacteroidales;f__[Barnesiellaceae] | 0.0010 | 0.0028 | 0.0010 | 0.0043 |
| k__Bacteria;p__Bacteroidetes;c__Bacteroidia;o__Bacteroidales;f__[Odoribacteraceae] | 0.0008 | 0.0027 | 0.0005 | 0.0019 |
| k__Bacteria;p__Bacteroidetes;c__Bacteroidia;o__Bacteroidales;f__[Paraprevotellaceae] | 0.0067 | 0.0200 | 0.0156 | 0.0317 |
| k__Bacteria;p__Bacteroidetes;c__Flavobacteriia;o__;f__ | 0.0000 | 0.0002 | 0.0000 | 0.0000 |
| k__Bacteria;p__Bacteroidetes;c__Flavobacteriia;o__Flavobacteriales;Other | 0.0000 | 0.0003 | 0.0000 | 0.0000 |
| k__Bacteria;p__Bacteroidetes;c__Flavobacteriia;o__Flavobacteriales;f__Cryomorphaceae | 0.0000 | 0.0001 | 0.0000 | 0.0000 |
| k__Bacteria;p__Bacteroidetes;c__Flavobacteriia;o__Flavobacteriales;f__Flavobacteriaceae | 0.0010 | 0.0078 | 0.0000 | 0.0000 |
| k__Bacteria;p__Bacteroidetes;c__Sphingobacteriia;o__Sphingobacteriales;f__ | 0.0001 | 0.0005 | 0.0000 | 0.0000 |
| k__Bacteria;p__Bacteroidetes;c__Sphingobacteriia;o__Sphingobacteriales;f__Chitinophagaceae | 0.0004 | 0.0027 | 0.0000 | 0.0000 |
| k__Bacteria;p__Chloroflexi;c__Anaerolineae;o__SBR1031;f__A4b | 0.0001 | 0.0008 | 0.0000 | 0.0000 |
| k__Bacteria;p__Cyanobacteria;c__4C0d-2;o__MLE1-12;f__ | 0.0000 | 0.0001 | 0.0000 | 0.0000 |
| k__Bacteria;p__Cyanobacteria;c__4C0d-2;o__YS2;f__ | 0.0002 | 0.0008 | 0.0000 | 0.0001 |
| k__Bacteria;p__Cyanobacteria;c__Chloroplast;Other;Other | 0.0001 | 0.0004 | 0.0000 | 0.0000 |
| k__Bacteria;p__Cyanobacteria;c__Chloroplast;o__Chlorophyta;f__Mamiellaceae | 0.0000 | 0.0004 | 0.0000 | 0.0000 |
| k__Bacteria;p__Cyanobacteria;c__Chloroplast;o__Streptophyta;f__ | 0.0000 | 0.0003 | 0.0001 | 0.0004 |
| k__Bacteria;p__Cyanobacteria;c__S15B-MN24;o__;f__ | 0.0001 | 0.0005 | 0.0002 | 0.0006 |
| k__Bacteria;p__Cyanobacteria;c__Synechococcophycideae;o__Synechococcales;f__Synechococcaceae | 0.0007 | 0.0021 | 0.0000 | 0.0001 |
| k__Bacteria;p__Elusimicrobia;c__Elusimicrobia;o__Elusimicrobiales;f__Elusimicrobiaceae | 0.0000 | 0.0000 | 0.0001 | 0.0002 |
| k__Bacteria;p__Firmicutes;Other;Other;Other | 0.0001 | 0.0005 | 0.0000 | 0.0001 |
| k__Bacteria;p__Firmicutes;c__Bacilli;Other;Other | 0.0010 | 0.0050 | 0.0008 | 0.0025 |
| k__Bacteria;p__Firmicutes;c__Bacilli;o__;f__ | 0.0000 | 0.0001 | 0.0000 | 0.0000 |
| k__Bacteria;p__Firmicutes;c__Bacilli;o__Bacillales;f__Alicyclobacillaceae | 0.0001 | 0.0007 | 0.0000 | 0.0000 |
| k__Bacteria;p__Firmicutes;c__Bacilli;o__Bacillales;f__Bacillaceae | 0.0000 | 0.0001 | 0.0000 | 0.0000 |
| k__Bacteria;p__Firmicutes;c__Bacilli;o__Bacillales;f__Paenibacillaceae | 0.0000 | 0.0004 | 0.0000 | 0.0000 |
| k__Bacteria;p__Firmicutes;c__Bacilli;o__Bacillales;f__Staphylococcaceae | 0.0003 | 0.0016 | 0.0000 | 0.0001 |
| k__Bacteria;p__Firmicutes;c__Bacilli;o__Gemellales;f__ | 0.0003 | 0.0010 | 0.0002 | 0.0008 |
| k__Bacteria;p__Firmicutes;c__Bacilli;o__Gemellales;f__Gemellaceae | 0.0004 | 0.0018 | 0.0000 | 0.0002 |
| k__Bacteria;p__Firmicutes;c__Bacilli;o__Lactobacillales;Other | 0.0002 | 0.0012 | 0.0000 | 0.0000 |
| k__Bacteria;p__Firmicutes;c__Bacilli;o__Lactobacillales;f__Aerococcaceae | 0.0001 | 0.0009 | 0.0000 | 0.0000 |
| k__Bacteria;p__Firmicutes;c__Bacilli;o__Lactobacillales;f__Enterococcaceae | 0.0081 | 0.0499 | 0.0026 | 0.0147 |
| k__Bacteria;p__Firmicutes;c__Bacilli;o__Lactobacillales;f__Lactobacillaceae | 0.0071 | 0.0543 | 0.0007 | 0.0040 |
| k__Bacteria;p__Firmicutes;c__Bacilli;o__Lactobacillales;f__Leuconostocaceae | 0.0001 | 0.0007 | 0.0000 | 0.0000 |
| k__Bacteria;p__Firmicutes;c__Bacilli;o__Lactobacillales;f__Streptococcaceae | 0.0187 | 0.0377 | 0.0253 | 0.1165 |
| k__Bacteria;p__Firmicutes;c__Bacilli;o__Turicibacterales;f__Turicibacteraceae | 0.0001 | 0.0003 | 0.0000 | 0.0003 |
| k__Bacteria;p__Firmicutes;c__Clostridia;Other;Other | 0.0019 | 0.0024 | 0.0013 | 0.0020 |
| k__Bacteria;p__Firmicutes;c__Clostridia;o__;f__ | 0.0013 | 0.0025 | 0.0025 | 0.0051 |
| k__Bacteria;p__Firmicutes;c__Clostridia;o__Clostridiales;Other | 0.0068 | 0.0081 | 0.0023 | 0.0031 |
| k__Bacteria;p__Firmicutes;c__Clostridia;o__Clostridiales;f__ | 0.0009 | 0.0020 | 0.0008 | 0.0028 |
| k__Bacteria;p__Firmicutes;c__Clostridia;o__Clostridiales;f__Catabacteriaceae | 0.0004 | 0.0018 | 0.0002 | 0.0009 |
| k__Bacteria;p__Firmicutes;c__Clostridia;o__Clostridiales;f__Clostridiaceae | 0.0018 | 0.0040 | 0.0028 | 0.0041 |
| k__Bacteria;p__Firmicutes;c__Clostridia;o__Clostridiales;f__Eubacteriaceae | 0.0001 | 0.0004 | 0.0001 | 0.0003 |
| k__Bacteria;p__Firmicutes;c__Clostridia;o__Clostridiales;f__Lachnospiraceae | 0.4259 | 0.1908 | 0.2232 | 0.2182 |
| k__Bacteria;p__Firmicutes;c__Clostridia;o__Clostridiales;f__Peptococcaceae | 0.0001 | 0.0003 | 0.0002 | 0.0005 |
| k__Bacteria;p__Firmicutes;c__Clostridia;o__Clostridiales;f__Peptostreptococcaceae | 0.0016 | 0.0031 | 0.0010 | 0.0032 |
| k__Bacteria;p__Firmicutes;c__Clostridia;o__Clostridiales;f__Ruminococcaceae | 0.0952 | 0.0778 | 0.0627 | 0.0830 |
| k__Bacteria;p__Firmicutes;c__Clostridia;o__Clostridiales;f__Veillonellaceae | 0.0073 | 0.0087 | 0.0048 | 0.0063 |
| k__Bacteria;p__Firmicutes;c__Clostridia;o__Coriobacteriales;f__ | 0.0000 | 0.0001 | 0.0000 | 0.0000 |
| k__Bacteria;p__Firmicutes;c__Clostridia;o__Coriobacteriales;f__Coriobacteriaceae | 0.0017 | 0.0030 | 0.0027 | 0.0043 |
| k__Bacteria;p__Firmicutes;c__Erysipelotrichi;o__Erysipelotrichales;f__Erysipelotrichaceae | 0.0433 | 0.1156 | 0.0125 | 0.0167 |
| k__Bacteria;p__Firmicutes;c__Erysipelotrichi;o__Erysipelotrichales;f__[Coprobacillaceae] | 0.0100 | 0.0163 | 0.0274 | 0.0547 |
| k__Bacteria;p__Fusobacteria;c__Fusobacteria;o__Fusobacteriales;f__Fusobacteriaceae | 0.0154 | 0.0664 | 0.0096 | 0.0285 |
| k__Bacteria;p__Fusobacteria;c__Fusobacteria;o__Fusobacteriales;f__Leptotrichiaceae | 0.0000 | 0.0000 | 0.0000 | 0.0001 |
| k__Bacteria;p__PAUC34f;c__;o__;f__ | 0.0000 | 0.0003 | 0.0000 | 0.0000 |
| k__Bacteria;p__Planctomycetes;c__Planctomycetia;o__Pirellulales;f__Pirellulaceae | 0.0000 | 0.0001 | 0.0000 | 0.0000 |
| k__Bacteria;p__Proteobacteria;Other;Other;Other | 0.0002 | 0.0009 | 0.0000 | 0.0000 |
| k__Bacteria;p__Proteobacteria;c__Alphaproteobacteria;o__Caulobacterales;f__Caulobacteraceae | 0.0000 | 0.0003 | 0.0000 | 0.0001 |
| k__Bacteria;p__Proteobacteria;c__Alphaproteobacteria;o__RF32;f__ | 0.0000 | 0.0003 | 0.0001 | 0.0004 |
| k__Bacteria;p__Proteobacteria;c__Alphaproteobacteria;o__Rhizobiales;f__Bradyrhizobiaceae | 0.0000 | 0.0003 | 0.0000 | 0.0001 |
| k__Bacteria;p__Proteobacteria;c__Alphaproteobacteria;o__Rhizobiales;f__Phyllobacteriaceae | 0.0000 | 0.0004 | 0.0000 | 0.0003 |
| k__Bacteria;p__Proteobacteria;c__Alphaproteobacteria;o__Rhodobacterales;f__Rhodobacteraceae | 0.0002 | 0.0016 | 0.0000 | 0.0000 |
| k__Bacteria;p__Proteobacteria;c__Alphaproteobacteria;o__Rhodospirillales;f__Rhodospirillaceae | 0.0000 | 0.0001 | 0.0000 | 0.0000 |
| k__Bacteria;p__Proteobacteria;c__Alphaproteobacteria;o__Rickettsiales;f__Pelagibacteraceae | 0.0002 | 0.0009 | 0.0000 | 0.0000 |
| k__Bacteria;p__Proteobacteria;c__Alphaproteobacteria;o__Sphingomonadales;f__Sphingomonadaceae | 0.0001 | 0.0008 | 0.0000 | 0.0000 |
| k__Bacteria;p__Proteobacteria;c__Betaproteobacteria;o__Burkholderiales;Other | 0.0000 | 0.0002 | 0.0001 | 0.0005 |
| k__Bacteria;p__Proteobacteria;c__Betaproteobacteria;o__Burkholderiales;f__Alcaligenaceae | 0.0083 | 0.0150 | 0.0092 | 0.0200 |
| k__Bacteria;p__Proteobacteria;c__Betaproteobacteria;o__Burkholderiales;f__Burkholderiaceae | 0.0002 | 0.0014 | 0.0000 | 0.0002 |
| k__Bacteria;p__Proteobacteria;c__Betaproteobacteria;o__Burkholderiales;f__Comamonadaceae | 0.0004 | 0.0019 | 0.0005 | 0.0019 |
| k__Bacteria;p__Proteobacteria;c__Betaproteobacteria;o__Burkholderiales;f__Oxalobacteraceae | 0.0070 | 0.0384 | 0.0294 | 0.1267 |
| k__Bacteria;p__Proteobacteria;c__Betaproteobacteria;o__Neisseriales;f__Neisseriaceae | 0.0003 | 0.0018 | 0.0007 | 0.0025 |
| k__Bacteria;p__Proteobacteria;c__Betaproteobacteria;o__Procabacteriales;f__Procabacteriaceae | 0.0001 | 0.0005 | 0.0000 | 0.0003 |
| k__Bacteria;p__Proteobacteria;c__Deltaproteobacteria;o__Bdellovibrionales;f__Bdellovibrionaceae | 0.0000 | 0.0003 | 0.0000 | 0.0000 |
| k__Bacteria;p__Proteobacteria;c__Deltaproteobacteria;o__Desulfovibrionales;f__Desulfovibrionaceae | 0.0003 | 0.0009 | 0.0002 | 0.0007 |
| k__Bacteria;p__Proteobacteria;c__Deltaproteobacteria;o__GMD14H09;f__ | 0.0000 | 0.0003 | 0.0000 | 0.0000 |
| k__Bacteria;p__Proteobacteria;c__Deltaproteobacteria;o__Myxococcales;f__ | 0.0000 | 0.0001 | 0.0000 | 0.0000 |
| k__Bacteria;p__Proteobacteria;c__Deltaproteobacteria;o__Myxococcales;f__0319-6G20 | 0.0000 | 0.0003 | 0.0000 | 0.0000 |
| k__Bacteria;p__Proteobacteria;c__Epsilonproteobacteria;o__Campylobacterales;Other | 0.0000 | 0.0000 | 0.0000 | 0.0001 |
| k__Bacteria;p__Proteobacteria;c__Epsilonproteobacteria;o__Campylobacterales;f__Campylobacteraceae | 0.0012 | 0.0065 | 0.0259 | 0.1136 |
| k__Bacteria;p__Proteobacteria;c__Epsilonproteobacteria;o__Campylobacterales;f__Helicobacteraceae | 0.0000 | 0.0000 | 0.0001 | 0.0004 |
| k__Bacteria;p__Proteobacteria;c__Gammaproteobacteria;Other;Other | 0.0000 | 0.0003 | 0.0000 | 0.0002 |
| k__Bacteria;p__Proteobacteria;c__Gammaproteobacteria;o__Aeromonadales;f__Succinivibrionaceae | 0.0000 | 0.0000 | 0.0001 | 0.0002 |
| k__Bacteria;p__Proteobacteria;c__Gammaproteobacteria;o__Alteromonadales;f__OM60 | 0.0000 | 0.0002 | 0.0000 | 0.0000 |
| k__Bacteria;p__Proteobacteria;c__Gammaproteobacteria;o__Chromatiales;f__ | 0.0000 | 0.0001 | 0.0000 | 0.0000 |
| k__Bacteria;p__Proteobacteria;c__Gammaproteobacteria;o__Enterobacteriales;f__Enterobacteriaceae | 0.0288 | 0.0888 | 0.2564 | 0.3566 |
| k__Bacteria;p__Proteobacteria;c__Gammaproteobacteria;o__Oceanospirillales;f__Halomonadaceae | 0.0001 | 0.0005 | 0.0000 | 0.0000 |
| k__Bacteria;p__Proteobacteria;c__Gammaproteobacteria;o__Pasteurellales;f__Pasteurellaceae | 0.0045 | 0.0095 | 0.0056 | 0.0112 |
| k__Bacteria;p__Proteobacteria;c__Gammaproteobacteria;o__Pseudomonadales;f__Moraxellaceae | 0.0002 | 0.0013 | 0.0001 | 0.0004 |
| k__Bacteria;p__Proteobacteria;c__Gammaproteobacteria;o__Pseudomonadales;f__Pseudomonadaceae | 0.0005 | 0.0015 | 0.0005 | 0.0015 |
| k__Bacteria;p__SBR1093;c__EC214;o__;f__ | 0.0000 | 0.0000 | 0.0000 | 0.0001 |
| k__Bacteria;p__Spirochaetes;c__[Brachyspirae];o__[Brachyspirales];f__Brachyspiraceae | 0.0000 | 0.0000 | 0.0738 | 0.2404 |
| k__Bacteria;p__Synergistetes;c__Synergistia;o__Synergistales;f__Synergistaceae | 0.0003 | 0.0022 | 0.0001 | 0.0003 |
| k__Bacteria;p__TM7;c__TM7-3;o__;f__ | 0.0000 | 0.0000 | 0.0000 | 0.0003 |
| k__Bacteria;p__Tenericutes;c__Mollicutes;o__Anaeroplasmatales;f__Anaeroplasmataceae | 0.0000 | 0.0000 | 0.0001 | 0.0005 |
| k__Bacteria;p__Tenericutes;c__Mollicutes;o__Mycoplasmatales;f__Mycoplasmataceae | 0.0000 | 0.0002 | 0.0000 | 0.0000 |
| k__Bacteria;p__Tenericutes;c__Mollicutes;o__RF39;f__ | 0.0001 | 0.0004 | 0.0000 | 0.0000 |
| k__Bacteria;p__Verrucomicrobia;c__Opitutae;o__[Cerasicoccales];f__[Cerasicoccaceae] | 0.0000 | 0.0000 | 0.0000 | 0.0003 |
| k__Bacteria;p__Verrucomicrobia;c__Verrucomicrobiae;o__Verrucomicrobiales;f__Verrucomicrobiaceae | 0.0083 | 0.0261 | 0.0002 | 0.0011 |
