# Supplementary material for: A Compositional Look at the Human Gastrointestinal Microbiome and Immune Activation Parameters in HIV Infected Subjects
Source: PLoS Pathog. 2014 Feb 20;10(2):e1003829. doi: 10.1371/journal.ppat.1003829 (PMC3930561; doi:10.1371/journal.ppat.1003829)
Supplement: Table S8 — Correlations between cytokines (Kendall's tau) used in CCA analysis. (DOCX) [file ppat.1003829.s025.docx]

**Table S8.** Correlations between cytokines (Kendall’s tau) used in CCA analysis

|  | **IL-6** | **TNF** | **LTA** | **sCD14** |
| --- | --- | --- | --- | --- |
| **IL-6** | 1 | 0.275 | 0.173 | 0.197 |
| **TNF** |  | 1 | 0.554 | 0.668 |
| **LTA** |  |  | 1 | 0.523 |
| **sCD14** |  |  |  | 1 |
